# Targeting ferroptosis regulators by natural products in colorectal cancer

**DOI:** 10.3389/fphar.2024.1374722

**Published:** 2024-05-27

**Authors:** Yiping Zhang, Jun Xie

**Affiliations:** ^1^ School of Life Sciences, Fudan University, Shanghai, China; ^2^ Wanchuanhui (Shanghai) Medical Technology Co., Ltd., Shanghai, China

**Keywords:** colorectal cancer, ferroptosis, natural products, ROS, iron

## Abstract

Colorectal cancer (CRC) poses a significant global health challenge, ranking as the third most diagnosed cancer and the second leading cause of cancer-related deaths. Despite advancements in treatment, challenges such as delayed diagnosis, multidrug resistance, and limited therapeutic effectiveness persist, emphasizing the need for innovative approaches. This review explores the potential of natural products, nutraceuticals, and phytochemicals for targeting ferroptosis-related regulators as a novel strategy in CRC. Ferroptosis, a form of regulated cell death characterized by iron-dependent lethal lipid peroxide accumulation, holds substantial importance in CRC progression and therapy resistance. Natural products, known for their diverse bioactive effects and favorable safety profiles, emerge as promising candidates to induce ferroptosis in CRC cells. Exploring amino acid, iron, lipid metabolism regulators, and oxidative stress regulators reveals promising avenues for inducing cell death in CRC. This comprehensive review provides insights into the multifaceted effects of natural products on proteins integral to ferroptosis regulation, including GPX4, SLC7A11, ACSL4, NCOA4, and HO-1. By elucidating the intricate mechanisms through which natural products modulate these proteins, this review lays the foundation for a promising therapeutic strategy in CRC.

## 1 Introduction

Colorectal cancer (CRC) represents a considerable worldwide health challenge, with an estimated 1.9 million new cases and 1 million deaths in 2020. While it is the third most frequently diagnosed cancer, it stands as the second leading cause of death. Developed countries experience notably higher incidence rates, a phenomenon associated with shifts in lifestyle and heightened socioeconomic development (Wang et al., 2020; Sung et al., 2021; Huang et al., 2023). Despite advancements in CRC treatment methods, the prognosis for patients remains challenging, particularly in the advanced stages of the disease. Delayed diagnosis at advanced stages, the development of acquired multidrug resistance, systemic toxicity from chemotherapy, tumor heterogeneity, and the limited effectiveness of current therapies contribute to the persistently poor prognosis (Wang et al., 2021; Liang W. et al., 2023; Kumar et al., 2023; Shen et al., 2023).

Natural products, comprising nutraceuticals and phytochemicals sourced from plants, have recently become a focal point in colon cancer research due to their potential for preventing and treating the disease (Sun et al., 2023). These compounds, offer diverse bioactive effects, such as antioxidative, anti-inflammatory, and anti-cancer properties, making them appealing candidates (Pan et al., 2022). Their limited side effects, favorable safety profile, and alignment with dietary influences on colorectal cancer risk contribute to their attractiveness. Additionally, the synergistic effects resulting from the combination of bioactive compounds within natural products and their global availability enhance their potential for cost-effective and widespread use (Islam et al., 2022).

Ferroptosis is a form of regulated cell death characterized by the iron-dependent accumulation of lethal lipid peroxides. In the context of CRC, ferroptosis holds substantial importance. Firstly, inducing ferroptosis in CRC cells is explored as a potential therapeutic strategy, targeting key modulators like glutathione (GSH), GPX4, and SLC7A11. Inhibition of ferroptosis may contribute to CRC progression and therapy resistance. Secondly, molecules associated with ferroptosis show promise as biomarkers for diagnosing and monitoring CRC, addressing challenges in delayed diagnosis. Thirdly, the therapeutic effect of targeting ferroptosis is an active area of research, aiming to overcome resistance to conventional chemotherapy in CRC. Lastly, understanding the role of ferroptosis in CRC progression provides crucial insights into disease dynamics, offering potential markers for early detection and prognosis prediction (Wang et al., 2022).

Current research explores compounds like cetuximab to modulate ferroptosis for improved chemotherapy outcomes. Challenges include the lack of standardized detection methods for ferroptosis and the validation of ferroptosis-related genes as CRC prognostic markers. Ongoing studies investigate the roles of proteins, clinically used drugs, and macrophages in tumor ferroptosis (Liang et al., 2022). Due to their diverse chemical composition, availability in nature, safety profiles, and ability to impact multiple cellular pathways, natural products can effectively trigger ferroptosis. These compounds, sourced from various origins, such as plants and herbs, exhibit a wide range of chemical structures and action mechanisms. Their multifaceted effects enable simultaneous influence on iron metabolism, lipid peroxidation, and antioxidant systems. Natural products often play a role in adjusting cellular equilibrium, interacting with signaling pathways related to both cell death and survival, and displaying antioxidant and anti-inflammatory characteristics. The historical utilization of certain natural products in traditional medicine, coupled with clinical evidence supporting their safety and effectiveness in treating cancers, lend credibility to their potential as agents inducing ferroptosis (Nie et al., 2023).

Natural products exert significant effects on proteins involved in ferroptosis. These proteins, integral to ferroptosis regulation, include but are not limited to GPX4, SLC7A11, and ACSL4. Natural compounds impact these enzymes through various mechanisms, such as inhibiting GPX4 to induce lipid peroxidation and cell death, modulating HO-1’s dual role in ferroptosis based on cellular iron and ROS levels, targeting NQO1 to trigger ferroptosis in cancer cells, and inhibiting NOX4 to resist ferroptosis. Additionally, natural products interact with regulators of lipid metabolism, like ACSL3 and ACSL4, influencing ferroptosis sensitivity. These findings highlight the diverse and complex ways in which natural products can modulate enzymes involved in ferroptosis, offering potential avenues for therapeutic interventions in cancers (Zuo et al., 2023).

Many articles have highlighted the significance of targeting ferroptosis for the treatment of colorectal cancer (Zhang et al., 2023a; Liang H. et al., 2023; Cheng et al., 2023; Liu et al., 2024). Therefore, this review aims to present a comprehensive perspective on the impact of natural products on proteins and pathways associated with ferroptosis in CRC (Table 1). It marks the first exploration of this approach as a promising strategy against CRC.

**TABLE 1:** Natural derived products in targeting ferroptosis regulator enzymes in colorectal cancer.

Natural product name	Source	Dose	Cell lines	Other targets	Signalling pathways	Effects	Model
GPX4							
Honokiol (Guo et al., 2021)	Magnolia officinalis	0.1–100 μM	SW48, HT29, LS174T, HCT116, HCT8, RKO, and SW480	—	Apoptotic Pathways	↓Cell viability and growth	*In Vivo* and *In Vitro*
						Change of mitochondrial morphology	
						↓Tumor volumes and weights	
Luteolin (Zheng et al., 2023)	Fruits, vegetables, and CHMs	1.56–50 μM	HCT116 and SW480 cells	HIC1	HIC1/GPX4	↓ Cell viability and proliferation	*In Vivo* and *In Vitro*
						↓Tumor volumes and weights	
Mollugin (Wang et al., 2023c)	Rubia cordifolia L. (Rubiaceae)	10–40 μM	SW620 cells and DLD-1 cells	IGF2BP3	IGF2BP3/GPX4	↓ Cell viability and proliferation	*In Vivo* and *In Vitro*
						↓Tumor volumes and weights	
3β-Hydroxy-12-oleanen-27-oic Acid (Tu et al., 2023)	Rhizome of Astilbe chinensis (Maxim.)	2.5–40 μM	HCT116	FDFT1	—	↓ Cell viability and proliferation	*In Vivo* and *In Vitro*
						↓Tumor volumes and weights	
2,3,5,4′-Tetrahydroxystilbene (TG1) (Tsai et al., 2023)	Polygonum multiflorum	10–100 μM	DLD-1, HCT116, and HT-29	FTH1	Autophagic pathways	↓ Cell viability and proliferation	*In Vivo* and *In Vitro*
						↓Tumor volumes and weights	
Bufotalin (Wu et al., 2023a)	Chansu	30–50 μg/mL	HCT116, NIH-3T3 and SW480	HSP70 and HIF-1	—	Excellent tumor-targeting ability	*In Vivo* and *In Vitro*
						↓Tumor volumes and weights	
						No obvious toxic or side effects on main organs	
Glycyrrhetinic Acid (Li et al., 2022b)	Licorice	30 and 60 µM	HT29, Caco-2, SW480	—	—	There is minimal drug toxicity of GCMNPs	*In Vivo* and *In Vitro*
						↓Tumor volumes and weights	
SLC7A11							
Curdione (Wang et al., 2023b)	Curcumae Rhizoma	12.5–50 μM	SW480 and CT26	SLC3A2 and GPX4	—	↓ Cell viability	*In Vivo* and *In Vitro*
						↓Tumor volumes and weights	
Pt3R5G (Han et al., 2022)	fruit of Lycium ruthenicum Murray	125–500 μg/mL	RKO and HCT116	GPX4, TFR1	Cell cycle, DNA replication, ferroptosis and p53	↓ Cell proliferation and migration	*In Vivo* and *In Vitro*
						↓Tumor volumes and weights	
Punicic Acid (Vermonden et al., 2021)	Pomegranate seed oil	14 µM	HCT-116 or FaDu cells	—	—	↓ Cell viability	*In Vitro*
IMCA (Zhang et al., 2020)	Sweet clover and tonka beans	12.5–200 μM	DLD-1 and HCT-116	GPX4	AMPK/mTOR	↓ Cell viability	*In Vivo* and *In Vitro*
						↓Tumor volumes and weight	
Vitamin D (Guo et al., 2023)	Fish, Egg, Cod Liver Oil	50 and 100 nM	HCT-116	GPX4 and COX-2	—	↓ Cell proliferation and viability	*In Vivo* and *In Vitro*
						↓Tumor volumes and weight	
Butyrate (He et al., 2023)	Bacterial fermentation of dietary fibers in the colon	0.5 mM	HCT116	cFOS	C-Fos/xCT	↓ Cell proliferation and viability	*In Vivo* and *In Vitro*
						↓Tumor volumes and weight	
Sodium Butyrate (NaB) (Bian et al., 2023)	Bacterial fermentation of dietary fibers in the colon	0.5–20 mM	HCT-116	CD44	CD44/SLC7A11	↓ Cell proliferation and viability	*In Vivo* and *In Vitro*
				GPX4		“NaB inhibit the CD44 and SLC7A11 in a murine CRC model”	
NRF2							
Lysionotin (Gao et al., 2022)	Lysionotus pauciflorus Maxim	5–60 μM	HCT116 and SW480	FTH1, GPX4	Keap1-Nrf2	↓ Cell proliferation, migration, and invasion	*In Vivo* and *In Vitro*
						↓Tumor volumes and weight	
Ginsenoside Rh3 (Wu et al., 2023b)	Hot-processed ginseng	10–160 μM	HT29, HCT116, SW620, DLD1 and RKO	xCT/SLC7A11 and GPX4	Stat3/p53/NRF2	↓ Cell proliferation and viability	*In Vivo* and *In Vitro*
						↓Tumor growth	
Tagitinin C (Wei et al., 2021)	Asteraceae	5–20 µM	HCT116	HO-1	PERK-Nrf2-HO-1	↓ Growth, colony formation, and cell migration	*in Vitro*
TF and TFRC							
Palmitic acid (Kuang et al., 2023)	Palm oil	120 μm	HT29, HCT116, SW480 and SW620	FPN	ER stress and CD36	↓ Cell proliferation and viability	*In Vivo* and *In Vitro*
						↓Tumor growth	
FTH							
β-elemene (Chen et al., 2020b)	Curcumae Rhizoma	125 μg/mL	HCT116, Lovo and CaCO2	GPX4, SLC7A11, and SLC40A1	TF/STEAP3	↓ Cell proliferation, migration, and invasion	*In Vivo* and *In Vitro*
						↓Tumor growth and lymph node metastasis	
HO-1							
Andrographolide (Sharma et al., 2020)	Andrographis paniculata	10–40 ng/μL	HCT116 and SW480	GCLC GCLM	β-catenin/Wnt	↓ Cell proliferation and viability	*In Vivo* and *In Vitro*
						↓Tumor growth	
Betula etnensis Raf. Extract (Malfa et al., 2019)	B. etnensis Raf. bark	5–500 μg/mL	CaCo2	γ-GCS	—	↓ Cell proliferation	*in Vitro*
NCOA4							
β-Lapachone (Zhao et al., 2024)	lapacho tree (Tabebuia avellanedae)	1–8 μM	SW620, SW480, and DLD-1	SLC7A11, GPX4, FTH1, TFRC	JNK pathway	↓ Cell proliferation and viability	*In Vivo* and *In Vitro*
						↓Tumor growth	
Puerarin (Lian et al., 2023)	Pueraria lobata (Willd.) Ohwi	5–30 μM	HT-29	GPX4	ATG5 (Autophagy)	↓ Cell proliferation and viability	*In Vitro*
Erianin (Miao et al., 2023)	Dendrobium species	50 and 100 nM	HCT116 and LoVo	FTH1, GPX4, ATG5	Autophagy	↓ Cell proliferation, migration, invasion and EMT	*In vivo* and *in Vitro*
						↓Tumor growth	
Ginsenoside Rh4 (Wu et al., 2022)	Ginseng	25–300 μM	HT29, HCT116, DLD1, and RKO	SLC7A11, GPX4, FTH1, DMT1	Autophagy	↓ Cell proliferation	*In Vivo* and *In Vitro*
						↓Tumor growth	
ACSL4							
Bromelain (Park et al., 2018)	Ananas comosus L. (Bromeliaceae)	50 μg/mL	Caco2, NCI-H508, HCT116, G13D, DLD1, and G12D	—	—	↓ Cell proliferation and viability	*In Vitro*
ALOX15							
Docosahexaenoic acid (Shan et al., 2022)	Fish oil	10 μM	HT-29	GPX4	Both ALOX5-dependent and ALOX5-independent pathways	↓ Cell viability	*In Vivo* and *In Vitro*
						↓Tumor size and weight	
AMPK							
Gambogenic acid (Ma et al., 2023)	Gamboge	0.5–2 μM	HCT116, HT29, DLD-1, HCT15, SW620, LOVO and RKO	SLC7A11/GPX4	BIP/PERK/eIF2A/ATF4	↓ Cell proliferation	*In Vivo* and *In Vitro*
						↓Tumor growth	
NaB (Wang et al., 2023e)	Intestinal bacteria	2 mM	HT29 or HCT116	SLC7A11, GPX4, ACSL,NRF2, p53, and ALOX12	mTOR pathway	↓ Cell viability	*In Vivo* and *In Vitro*
						↓Colony formation	
mTOR							
Curcumin (Chen et al., 2023b)	Curcuma Longa	5–100 μM	HCT-8	SLC7A11, GPX4	PI3K/Akt/mTOR	↓ Cell proliferation	*In Vitro*
AMPK/mTOR							
Osthole (Zhou et al., 2023)	Cnidium spp. and Apiaceous plants	25–100 μM	HCT116 and SW480	SLC7A11, GPX4, NCOA4, HO-1, and TFR	Akt pathway	↓ Cell proliferation	*In Vivo* and *In Vitro*
						↓Tumor volumes and weight	
FSP1							
Curcumin and Andrographis (Miyazaki et al., 2023)	Curcuma Longa/andrographis paniculate	Variable	SW480 and HCT116	GPX4	—	↓ Cell proliferation and invasion	*In Vivo* and *In Vitro*
						↓Tumor growth	
Timosaponin AIII (Shen et al., 2024)	Anemarrhena asphodeloides	10 µM	CT116 and SW480	GPX4	—	↓ Cell viability	*In Vivo* and *In Vitro*
						↓Tumor growth and volume	

## 2 Natural products targeting system Xc^−^/GPX4 axis in CRC

**FIGURE 1:**
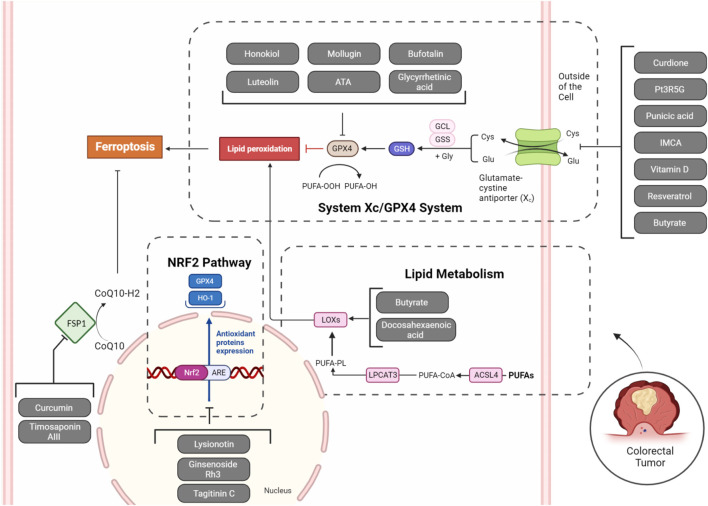
Natural products targeting System Xc^−^/GPX4 axis, lipid metabolism (ALOXs), NRF2 pathway, and FSP1 in ferroptosis of CRC cells. Inhibition of System Xc^−^ (SLC7A11), GPX4, NRF2, and FSP1, along with the activation of ALOXs by natural products, can collectively induce ferroptosis in tumoral cells.

The metabolism of amino acids plays a vital role in the occurrence of ferroptosis. Various amino acids, including cysteine, glutamine, and glycine, are intricately involved in modulating ferroptosis. For instance, cysteine is essential for the synthesis of the antioxidant GSH, and its depletion leads to increased reactive oxygen species (ROS) and induces ferroptosis. Glutamine, a key amino acid in cancer metabolism, fuels ferroptosis through glutaminolysis (Yang et al., 2022). Glutathione peroxidase 4 (GPX4) and System Xc^−^ play important roles in amino acid metabolism, particularly in redox regulation and antioxidant defense. GPX4 is a selenium-containing enzyme that contributes to cellular protection against oxidative stress by reducing lipid hydroperoxides, hindering the exacerbation of lipid peroxidation. This process is crucial for maintaining membrane integrity and preventing ferroptosis. Conversely, SLC7A11 is responsible for encoding the xCT subunit of the system Xc^−^ cystine/glutamate antiporter. This system facilitates the cellular import of cystine while releasing glutamate in exchange. Cystine serves as a precursor for the production of glutathione, a significant antioxidant within cells. Elevated SLC7A11 expression is linked to improved cellular antioxidant capability, as it facilitates the synthesis of GSH. Together, GPX4 and SLC7A11 contribute to the cellular defense against oxidative stress by regulating the levels of antioxidant molecules and protecting cells from harmful reactive oxygen species (Forcina and Dixon, 2019; Koppula et al., 2021). Herein, we introduce many natural products that can target them in CRC (Figure 1).

### 2.1 System Xc^−^ and GPX4 inhibitors

In the realm of ferroptosis, inhibiting xCT in System Xc^−^ induces cystine scarcity, diminishing GSH levels, and heightening cellular vulnerability to lipid peroxidation-induced demise. Notably, tumor cells, especially those harboring specific oncogenic mutations, display heightened susceptibility to ferroptosis, positioning xCT as a compelling target for cancer therapy. The intricate regulatory mechanisms governing xCT, spanning transcriptional, epigenetic, and post-translational controls, underscore the nuanced role of xCT within System Xc^−^ in shaping cellular redox dynamics, carrying significant implications for cancer treatment, particularly in the context of drug-resistant tumors (Li F-J. et al., 2022). The principal subunit of System Xc^−^, SLC7A11, may undergo upregulation in colorectal cancers potentially due to m6A modification by ALKBH5 (Luo et al., 2023), and Xc^−^ inhibitor drugs such as sulfasalazine have shown chemosensitizing effects to cisplatin in colorectal cancer (Ma et al., 2015). Similarly, GPX4 is a vital enzyme that protects cells from oxidative damage by inhibiting lipid peroxidation. Its unique ability to reduce larger organic peroxides, including polyunsaturated lipids, plays a key role in preventing the accumulation of harmful lipid-derived peroxides that can damage cell membranes. GPX4, regulated by various transcription factors and selenium availability, catalyzes the reduction of peroxides using glutathione, converting them into non-toxic lipid alcohols. The protein’s structure involves a catalytic tetrad with selenocysteine and conserved residues. GPX4 is crucial for embryonic development, and mutations in its gene are linked to a rare lethal disease. Additionally, GPX4 prevents ferroptosis, making it a potential target for therapeutic interventions, including cancer treatment (Forcina and Dixon, 2019). Due to frequent m6A and m5c modification of GPX4 in CRC, it is highly overexpressed (Chen B. et al., 2023; Zhang G. et al., 2023), leading to anti-cancer immunity (Chen B. et al., 2023), radiation resistance (Zhang et al., 2023c), and chemoresistance (Wang L. et al., 2023). The increased presence of GPX4 is detected in CRC persister cells resulting from exposure to anti-colorectal cancer drugs like 5-FU and AZ628. The elevation of GPX4 in these cells enhances their ability to survive various treatments, including chemotherapy. The heightened GPX4 expression is linked to greater resistance against oxidative stress caused by the drugs. As a result, the inhibition of GPX4 through GPX4 inhibitors like RSL3 specifically triggers ferroptotic cell death in CRC persister cells, rendering them more susceptible to this controlled form of cell death (Zhang X. et al., 2022). Thus, inhibiting their activity with natural products, which potentially have fewer toxic effects on normal cell lines, should be considered. Up to now, many studies have evaluated the potential utility of natural SLC7A11 and GPX4 inhibitors in CRC (Figure 1).

#### 2.1.1 Curdione

The potential of curdione, a significant sesquiterpene found in Curcumae Rhizoma, a traditional Chinese medicine, to combat CRC by inducing ferroptosis has been emphasized. Curdione exhibits inhibitory effects on murine-derived CRC cell lines in a manner dependent on dosage, leading to a reduction in cell viability. Treatment with curdione also results in a significant rise in intracellular ROS and encourages lipid peroxidation, indicating the occurrence of ferroptosis. Additionally, in CRC mice, the administration of curdione leads to tumor suppression and alterations in iron ions, MDA, LPO, and GSH levels, providing further evidence of its potential as a therapeutic agent. In terms of mechanism, curdione elevates the expression of METTL14, a methyltransferase, and triggers m6A modification in the Xc system, initiating ferroptosis. Knockdown experiments validate the role of METTL14 in curdione-induced ferroptosis. Research indicates that curdione interacts with key enzymes that regulate ferroptosis, such as GPX4 and ACSL4. By modulating the activity of GPX4, curdione may reduce the enzyme’s ability to detoxify lipid peroxides, thus promoting lipid peroxidation and cell death. Additionally, curdione’s effect on ACSL4 could influence the lipid composition of membranes, making them more susceptible to oxidative damage. Curdione’s ability to inhibit CRC cell viability, induce ferroptosis, modulate protein expression related to ferroptosis and RNA modification, and reduce tumor growth in CRC xenograft models demonstrate its multifaceted anti-cancer properties. Importantly, curdione’s specificity in inducing ferroptosis independent of apoptosis offers a distinct advantage, potentially complementing existing treatment strategies. These preclinical results provide a strong rationale for further clinical investigation of curdione in CRC patients, aiming to validate its efficacy and safety as a novel therapeutic approach. If successful, curdione could offer a valuable addition to the arsenal of CRC treatment options, particularly for patients who may not respond optimally to current therapies or for whom resistance has developed (Wang F. et al., 2023). However, the safety profile of curdione remains unexplored and necessitates further investigation to elucidate potential toxicities.

#### 2.1.2 Pt3R5G

Pt3R5G is a flavonoid compound isolated from Lycium ruthenicum Murray with potent antioxidant activity. Pt3R5G induces ferroptosis in CRC, particularly in RKO cells, by causing mitochondrial morphological changes. Pt3R5G interacts with ferroptotic proteins in colorectal cancer cells, leading to significant downstream effects on cellular pathways. Firstly, Pt3R5G treatment downregulates the expression of SLC7A11, which results in decreased cystine uptake and intracellular glutathione synthesis. Consequently, this leads to reduced cellular antioxidant capacity, making cells more susceptible to lipid peroxidation. Pt3R5G-induced lipid peroxidation results in the accumulation of lipid peroxides, such as malondialdehyde (MDA), and ROS. These changes in lipid metabolism and redox homeostasis contribute to the induction of ferroptosis. Furthermore, Pt3R5G treatment alters the expression of downstream proteins involved in ferroptosis regulation, such as increasing the expression of transferrin receptor 1 (TFR1) and decreasing the expression of GPX4. Additionally, Pt3R5G affects various cellular pathways, including the cell cycle, DNA replication, and apoptotic signaling, through its modulation of ferroptosis-related processes. This comprehensive understanding of Pt3R5G’s interactions with ferroptotic enzymes and its downstream effects on cellular pathways provides valuable insights into its potential as a therapeutic agent for colorectal cancer. The identified molecular alterations contribute to the observed anti-tumor effects of Pt3R5G, inhibiting cell proliferation and causing cell cycle arrest and apoptosis. *In vivo* studies using xenograft mice support Pt3R5G’s ability to suppress the growth of colon cancer cells without adverse effects on mouse weight. The *in vitro* and *in vivo* studies of Pt3R5G presented here offer promising avenues for its translation into clinical applications for colorectal cancer treatment. *In vitro* experiments demonstrated Pt3R5G’s potent inhibitory effects on colorectal cancer cell proliferation through induction of ferroptosis, evidenced by altered expression of key ferroptotic enzymes and downstream signaling proteins. These findings suggest Pt3R5G as a potential therapeutic agent for targeting ferroptosis in colorectal cancer cells. Furthermore, *in vivo* xenograft studies corroborated these findings, showing significant suppression of tumor growth without adverse effects on mouse weight at appropriate doses. The downregulation of SLC7A11, a pivotal regulator of ferroptosis, further underscores Pt3R5G’s potential clinical relevance. Using the MTT assay to evaluate cell viability, the findings revealed no notable variance in cell proliferation between untreated normal cells and those exposed to Pt3R5G at either concentration. This implies that Pt3R5G does not pose detrimental effects on the viability of normal cells, suggesting a potentially positive safety profile in non-cancerous cell populations (Han et al., 2022).

#### 2.1.3 Punicic acid

Punicic acid (PunA) is a polyunsaturated fatty acid found in pomegranate seed oil. PunA, a conjugated linolenic acid (CLnA) isomer, exhibits distinct cytotoxic effects compared to dihydroxy CLnA (DHA) on carcinoma cells. The natural product PunA inhibits GPX4 and induces lipid peroxidation, leading to ferroptotic cell death in CRC cells. PunA triggers ferroptosis by promoting the accumulation of lipid hydroperoxides, which is inhibited by ferroptosis inhibitors such as ferrostatin-1 and α-tocopherol. This accumulation of lipid hydroperoxides ultimately leads to cell death through ferroptotic pathways. Similarly, DHA, a PUFA, induces lipid peroxidation and ferroptosis in carcinoma cells. The downstream effects on cellular pathways involve the disruption of lipid homeostasis, mitochondrial dysfunction, and oxidative stress, culminating in ferroptotic cell death. Unlike DHA, PunA shows cytotoxicity against HCT-116 and FaDu carcinoma cells at micromolar doses, leading to a significant decrease in cell viability. Notably, PunA triggers ferroptosis in carcinoma cells, as evidenced by the inhibition of its cytotoxic effects by ferroptosis inhibitors such as ferrostatin-1 and α-tocopherol. This is in contrast to DHA, which does not induce ferroptosis in these cells. Furthermore, combining PunA with DHA enhances the cytotoxic effects on carcinoma cells, suggesting potential synergistic interactions between the two fatty acids. The *in vitro* and *in vivo* studies investigating the cytotoxic effects of PunA and DHA on carcinoma cells offer promising insights into potential clinical applications for cancer treatment. These findings suggest that these natural products could be developed into therapeutic agents targeting ferroptosis, a novel cell death mechanism in cancer cells. However, translating these preclinical findings into clinical practice requires rigorous evaluation through human trials. While there is a lack of ongoing or planned clinical trials specifically focused on PunA and DHA in cancer treatment, the promising results from preclinical studies warrant further investigation in human patients (Vermonden et al., 2021). The safety profiles of PunA and DHA are crucial considerations for their potential clinical applications. While PunA and DHA have demonstrated promising anti-cancer effects in preclinical studies, their safety profiles need to be thoroughly evaluated, particularly regarding potential adverse effects and toxicity in humans.

#### 2.1.4 IMCA

The potential anti-CRC effects of 2-Imino-6-methoxy-2H-chromene-3-carbothioamide (IMCA), a benzopyran derivative, have been highlighted by Zhang et al. for the first time. Firstly, IMCA downregulates the expression of SLC7A11, which leads to reduced uptake of cystine and subsequent depletion of GSH. This disruption in antioxidant defense mechanisms increases intracellular ROS levels. Additionally, IMCA promotes the phosphorylation of AMPK, which inhibits the mTOR pathway, further suppressing SLC7A11 expression. Consequently, the decreased availability of cystine and GSH, along with the inhibition of mTOR signaling, collectively contribute to the induction of ferroptosis in CRC cells. Furthermore, overexpression of SLC7A11 rescues the ferroptotic effects of IMCA, highlighting the pivotal role of SLC7A11 in mediating IMCA-induced ferroptosis. IMCA demonstrates significant efficacy in reducing CRC cell viability both *in vitro* and *in vivo* without causing significant toxicity to healthy tissues. These findings suggest that IMCA could serve as a potential candidate for further preclinical and clinical development in CRC treatment, potentially offering a more effective and safer alternative to conventional therapies. Further clinical studies are warranted to validate its efficacy and safety profile in human patients to translate these preclinical findings into tangible clinical applications for improving CRC treatment outcomes (Zhang et al., 2020).

#### 2.1.5 Vitamin D

The meta-analysis of randomized controlled trials demonstrated lower cancer mortality in populations receiving vitamin D supplementation in patients with CRC (Boakye et al., 2021). The molecular effects of vitamin D in CRC in a study were attributed to its capability to induce ferroptosis. The biologically active form of vitamin D, known as 1α,25-(OH)2D3, has demonstrated the ability to impede tumor growth and act as a transcriptional regulator via the vitamin D receptor (VDR). The study explored the intricate interaction between vitamin D (VD) and ferroptosis regulators in colorectal cancer stem cells (CCSCs), shedding light on its therapeutic potential. VD treatment was found to induce ferroptosis in CCSCs by downregulating the expression of SLC7A11. This downregulation led to reduced uptake of cystine and subsequent depletion of GSH, resulting in increased ROS levels and lipid peroxidation. Furthermore, VD treatment altered the expression of ferroptosis-related markers, such as GPX4 and COX2, further contributing to the induction of ferroptosis. Importantly, overexpression of SLC7A11 rescued VD-induced ferroptosis, highlighting the central role of SLC7A11 in mediating the effects of VD on ferroptosis in CCSCs.The observed inhibition of self-renewal and proliferation of CCSCs, along with enhanced sensitivity to chemotherapy upon VD treatment, suggests its potential utility as an adjuvant therapy to standard treatment regimens for colorectal cancer. Moreover, the ability of VD to selectively target CCSCs through ferroptosis induction underscores its potential as a targeted therapy to eradicate chemotherapy-resistant cancer cells and prevent tumor recurrence (Guo et al., 2023). In clinical trials involving patients with CRC, vitamin D supplementation has demonstrated a favorable safety profile. Specifically, in the SUNSHINE trial, which investigated high-dose vitamin D3 supplementation (4000 IU/day) alongside standard chemotherapy in metastatic CRC patients, no added toxicity was observed compared to standard-dose vitamin D3 (400 IU/day). Furthermore, vitamin D3 supplementation did not result in any significant adverse effects in terms of patient tolerance or treatment-related complications. This suggests that vitamin D supplementation, even at higher doses, is well-tolerated and safe for use in CRC patients undergoing chemotherapy. However, ongoing research, including the SOLARIS trial, continues to evaluate the safety and efficacy of vitamin D supplementation in CRC treatment protocols to provide comprehensive insights into its clinical utility and safety profile (Guo et al., 2023).

#### 2.1.6 Resveratrol

In recent years, researchers have investigated the potential of Resveratrol (RSV), a traditional Chinese medicine chemical monomer, in treating CRC. RSV exhibits anti-tumor effects by inducing ferroptosis. However, the limited bioavailability of RSV hampers its *in vivo* application. To overcome this challenge, researchers developed RSV-loaded nanoparticles coated with erythrocyte membranes (RSV-NPs@RBCm), employing biomimetic and drug delivery strategies. RSV NPs interact with ferroptosis regulators through multiple mechanisms, as elucidated by the study. Firstly, RSV NPs induce ferroptosis in colorectal cancer cells by triggering mitochondrial changes, such as mitochondrial disintegration and cristae reduction, which are characteristic of ferroptosis. Additionally, RSV NPs lead to the accumulation of intracellular iron (Fe^2+^), a hallmark of ferroptosis, as demonstrated by decreased fluorescence intensity of Phen Green SK, a heavy metal indicator. This accumulation is crucial for the initiation of the Fenton reaction, contributing to lipid peroxidation and, ultimately cell death. Furthermore, RSV NPs downregulate the expression of key ferroptosis-related proteins, including SLC7A11 and GPX4, which play pivotal roles in regulating intracellular glutathione levels and lipid peroxidation, respectively. These interactions collectively underscore the ability of RSV NPs to modulate ferroptosis pathways, highlighting their potential as effective agents for colorectal cancer treatment. The safety assessments conducted on RSV NPs demonstrated their excellent safety profile, both *in vitro* and *in vivo*. *In vitro* experiments revealed minimal toxicity of blank nanoparticles (NPs@RBCm) even at elevated concentrations (up to 400 μg/mL) in normal colorectal epithelial cell lines (NCM460), indicating remarkable biocompatibility. Additionally, RSV NPs showed negligible cytotoxicity in cancer cell lines HT29 and HCT116. *In vivo* investigations confirmed the safety of RSV NPs, as evidenced by the absence of adverse effects on body weight, tumor volume, and organ histopathology in treated mice compared to controls. Histological examination of major organs revealed no abnormalities, highlighting the favorable safety profile of RSV NPs.Moreover, the RSV NPs exhibit excellent biocompatibility, stability, and prolonged circulation time owing to their erythrocyte membrane coating, which enhances tumor targeting and penetration. Co-administration with iRGD peptide further improves tumor accumulation and efficacy. These results suggest that RSV NPs hold promise as a safe and effective therapeutic strategy for colorectal cancer, with the potential to address the limitations of conventional chemotherapy, offering new avenues for personalized and targeted cancer treatment in clinical settings. (Zhang Z. et al., 2022).

#### 2.1.7 Butyrate

The multifaceted roles of butyrate, a short-chain fatty acid produced by colonic gut bacteria, has been highlighted. Butyrate plays a crucial role in maintaining colonic health by serving as a major energy source for epithelial cells, promoting mucosal thickness, and enhancing the integrity of the intestinal barrier. Importantly, butyrate exhibits antineoplastic effects, inhibiting the proliferation of cancer cells through the modulation of signaling pathways (Hajjar et al., 2021). Recent research provides insights into how butyrate effectively tackles ferroptosis resistance in CRC by inducing c-Fos-dependent xCT suppression. Butyrate, a prevalent short-chain fatty acid in the colon, sensitizes CRC cells to ferroptosis by significantly reducing xCT. This reduction is attributed to butyrate’s inhibition of histone deacetylase (HDAC), specifically targeting class I HDACs. Additionally, a connection between butyrate-induced xCT suppression and c-Fos, a transcription factor whose expression rises with butyrate treatment, was established. Acting as an HDAC inhibitor, butyrate triggers changes in chromatin accessibility, leading to increased c-Fos expression. C-Fos, in turn, transcriptionally inhibits xCT expression, forming a crucial link in reversing ferroptosis resistance. This c-Fos-mediated xCT inhibition is validated *in vivo*, showing that butyrate administration enhances c-Fos expression in CRC tumors, correlating with decreased xCT expression and heightened ferroptosis (He et al., 2023). Importantly, elevated levels of xCT in CRC tissues correlate with decreased fecal butyrate concentration, suggesting a reciprocal relationship between butyrate production and xCT expression. This implies that butyrate may serve as a crucial determinant of ferroptosis sensitivity in CRC patients. Moreover, butyrate enhances the pro-ferroptotic effects of traditional chemotherapy drugs like oxaliplatin, indicating its potential as a sensitizer for improving the efficacy of existing CRC treatments. In a similar study by Bian et al., the potential of sodium butyrate (NaB) in inducing ferroptosis in CRC cells has been investigated. The results revealed that NaB induces cell death in CRC cells through both apoptosis and ferroptosis, selectively affecting cancer cells. A ferroptosis-related gene analysis identifies CD44 and SLC7A11 as potential effectors of NaB-induced ferroptosis. The study further confirms the upregulation of CD44 and SLC7A11 in human CRC and their correlation with poor prognosis. NaB is shown to inhibit CD44 and SLC7A11 expression both *in vitro* and in a murine CRC model. CD44 overexpression experiments validate its role in NaB-induced ferroptosis, and a synergistic effect is observed when NaB is combined with the ferroptosis-inducing agent Erastin. While NaB at high concentrations or prolonged exposure may induce apoptosis in normal intestinal epithelial cells, optimizing its concentration and exposure time is crucial to enhance its antitumor effect and minimize effects on normal colon cells. Erastin, a classic ferroptosis-inducing agent, showed minimal impact on cell viability at 10 μM concentration, but in combination with NaB, cell viability decreased significantly with increasing NaB concentration, accompanied by enhanced cytotoxicity (Bian et al., 2023).

#### 2.1.8 Honokiol

Honokiol (HNK), a natural compound extracted from Magnolia officinalis, demonstrates strong anticancer effects in CRC cells by initiating ferroptosis. HNK reduces the viability of CRC cells in a dose-dependent manner, accompanied by an increase in levels of ROS and changes in mitochondrial structure characteristic of ferroptosis. The compound disrupts the balance of iron in cells by boosting transferrin levels and diminishing ferroportin levels, leading to an accumulation of ferrous iron (Fe^2+^) and ROS. Specifically, HNK reduces cell viability by inhibiting GPX4, leading to the accumulation of ROS and Fe^2+^ ions. Notably, HNK does not affect system Xc^−^, the cystine/glutamate antiporter. This interaction triggers downstream effects on cellular pathways associated with ferroptosis, including alterations in mitochondrial morphology, such as increased membrane density and mitochondrial ridge shrinkage, indicative of ferroptotic changes. Moreover, HNK’s antitumor effects are validated in xenograft models, where GPX4 overexpression attenuates its efficacy. These findings suggest that HNK could serve as a therapeutic agent for CC by targeting ferroptosis-related pathways. Translating these preclinical results to clinical settings may involve exploring HNK as a potential adjuvant therapy or investigating its efficacy in combination with existing treatments for CC. Additionally, further clinical studies are warranted to evaluate HNK’s safety, pharmacokinetics, and efficacy in human patients, with the ultimate goal of improving CC treatment outcomes (Guo et al., 2021). However, the potential toxic effects of HNK on normal cells warrant further investigation.

#### 2.1.9 Luteolin

Luteolin, a natural flavonoid found in fruits and vegetables, exhibits diverse biological activities, including anti-tumor effects. In a study focused on CRC, luteolin, when combined with the ferroptosis-inducing agent erastin, demonstrates a significant inhibitory impact on CRC cell viability and proliferation. Studies have shown that erastin is more effective in inducing ferroptosis in certain cancer cell lines derived from non-epithelial tissues, while colon cancer cells may exhibit resistance to erastin-induced ferroptosis. In contrast, luteolin appears to sensitize colon cancer cells to ferroptosis, potentially overcoming this resistance. Therefore, while both luteolin and erastin induce ferroptosis, luteolin’s mechanism of action and efficacy may offer complementary or synergistic effects in the treatment of colon cancer. It primarily targets the Keap1/Nrf2 signaling pathway, suppressing Nrf2 activation and impairing the antioxidant defense system. This disruption leads to an accumulation of ROS within the cell. Concurrently, luteolin downregulates GPX4, a crucial enzyme in mitigating lipid peroxidation-induced cell death. With GPX4 inhibited, lipid peroxidation levels rise, compromising cell membrane integrity. The combination of increased ROS and lipid peroxidation induces ferroptosis, a form of programmed cell death characterized by iron-dependent lipid damage and subsequent cell demise. Moreover, luteolin may influence other pathways related to inflammation and cell proliferation, further contributing to its anticancer properties. Additionally, the regulatory protein hypermethylated in cancer 1 (HIC1) is identified as playing a role, with luteolin and erastin cotreatment upregulating HIC1 expression. This research reveals the synergistic anticancer effects of luteolin and erastin in CRC, implicating ferroptosis induction through modulation of GPX4 and HIC1 (Zheng et al., 2023). Nevertheless, further exploration is needed to assess the potential adverse impacts of HNK on healthy cells. The findings from both *in vitro* and *in vivo* studies suggest that luteolin holds promise for clinical applications in treating colon cancer, indicating potential translational significance.

#### 2.1.10 Mollugin

Mollugin, a compound found in Rubia cordifolia L., has exhibited diverse bioactive properties and demonstrated antitumor effects in various cancers. In the context of CRC, mollugin was examined for its potential to inhibit cell growth and induce ferroptosis in CRC cells. Mollugin effectively reduced cell viability and proliferation in CRC cells in laboratory settings. Mollugin’s inhibition of GPX4 expression is mediated by downregulating insulin-like growth factor 2 mRNA-binding protein 3 (IGF2BP3), which stabilizes GPX4 mRNA. This downregulation of GPX4 leads to increased levels of ROS and lipid peroxidation, hallmarks of ferroptosis. Additionally, mollugin treatment elevates Fe^2+^ levels and reduces GSH levels, further promoting ferroptosis. *In vivo* studies confirm mollugin’s ability to suppress tumor growth by inhibiting the IGF2BP3/GPX4 axis, underscoring its potential as a therapeutic agent for CRC by targeting ferroptosis regulators and associated pathways. These preclinical studies provide a strong rationale for further clinical development of mollugin as a potential therapy for CRC patients. However, additional investigation is required to evaluate the potential negative effects of HNK on normal cells (Wang W. et al., 2023).

#### 2.1.11 ATA

ATA (3β-hydroxy-12-oleanen-27-oic acid) is a cytotoxic compound derived from the rhizome of Astilbe chinensis, showing promise as an antitumor agent against CRC cells. A study has highlighted ATA’s ability to induce various forms of cell death, including apoptosis, autophagy, and ferroptosis, with a particular focus on ferroptosis induction. The key mechanism involves the inhibition of FDFT1, a protein associated with cholesterol biosynthesis, which is identified as a crucial mediator of ATA’s cytotoxic effects. Additionally, increased intracellular ROS levels and the downregulation of anti-ferroptotic protein GPX4 suggest a role for lipid peroxidation in the ferroptotic process. *In vivo* studies using xenograft mouse models further confirm ATA’s efficacy in inhibiting tumor growth and modulating ferroptosis-associated proteins. While inhibiting the proliferation of various cancer cell lines, including CRC, ATA exhibits minimal cytotoxic effects on normal human cells such as human umbilical vein endothelial cells (HUVECs) and lung fibroblasts. This selective cytotoxicity is attributed to ATA’s ability to induce apoptosis, autophagy, and ferroptosis specifically in cancer cells, while sparing normal cells. Furthermore, ATA’s distinctive antitumor mechanism, involving interactions with key regulators like GPX4, leads to dysregulation of cellular pathways associated with cancer progression, thus amplifying its cytotoxic effects against cancer cells while minimizing harm to normal cells. These findings suggest that ATA could be developed as a novel treatment option for CRC, offering improved clinical outcomes with reduced side effects compared to conventional therapies. Further clinical studies are warranted to validate its efficacy and safety for potential translation into clinical applications (Tu et al., 2023).

#### 2.1.12 TG1

TG1, a derivative of THSG sourced from Polygonum multiflorum, presents notable anticancer effects on CRC cells, showcasing dose-dependent cytotoxicity, reduced tumor volume in xenograft models, and induction of apoptosis and autophagy. Mechanistically, TG1 interacts with targeted enzymes involved in ferroptosis, a novel programmed cell death pathway characterized by lipid peroxidation. Specifically, TG1 downregulates ferroptosis suppressor genes, such as GPX4 and heat shock protein family B member 1 (HSPB1), while upregulating ferroptosis driver genes. This dysregulation of ferroptosis-related gene expression leads to the accumulation of iron-dependent lipid peroxides, ultimately triggering ferroptotic cell death in CRC cells. Additionally, TG1 modulates various cellular pathways implicated in cancer progression, including apoptosis, autophagy, and MYC signaling, further contributing to its anticancer effects. Importantly, TG1 demonstrates low toxicity and inhibits tumor growth *in vivo*, making it a promising candidate for further preclinical and clinical evaluation (Tsai et al., 2023).

#### 2.1.13 Bufotalin

Naturally derived products have been employed in nanoparticle forms to trigger ferroptosis by inhibiting GPX4 in colorectal cancer. In a study by Wu et al., a novel nanosystem named PCM (Bufotalin–Prussian blue biomimetic nanoparticle) was developed for chemo-photothermal therapy against CRC. Bufotalin (CS-5), a natural compound derived from traditional Chinese medicine, was loaded into Prussian blue nanoparticles (PB) to form PC, and then combined with a hybridized erythrocyte–tumor membrane to create PCM. This nanosystem exhibited outstanding stability, high drug-loading efficiency, and controlled release. PCM demonstrated tumor-targeting properties and prolonged circulation time owing to the hybrid membrane. PC interacts with targeted enzymes and induces downstream effects on cellular pathways through a multifaceted mechanism. Firstly, PC contains bufotalin, which has been shown to downregulate the expression of GPX4, an enzyme crucial for protecting cells against lipid peroxidation. By inhibiting GPX4, PC disrupts the cellular antioxidant defense mechanism, leading to the accumulation of toxic lipid ROS. This accumulation of ROS, coupled with the release of iron and ferrous ions from Prussian blue, triggers a form of iron-dependent cell death known as ferroptosis. Moreover, the hybridized erythrocyte-tumor membrane coating of PCM provides tumor-targeting properties and prolonged blood circulation time, enhancing its accumulation at the tumor site. Additionally, PC has been shown to downregulate the expression of HSP70 and hypoxia-inducible factor 1-alpha (HIF-1α), further sensitizing cancer cells to chemotherapy and photothermal therapy. The combined effects of CS-5-induced apoptosis and PB-induced ferroptosis led to a potent chemo-photothermal therapy, resulting in significant inhibition of tumor growth in CRC mouse models. Furthermore, PCM exhibited high biological safety and potential modifications in gut microbiota, highlighting its potential for clinical applications in the treatment of colorectal cancer. The cytotoxicity analysis conducted on various cell lines indicated high cell viability even at relatively high concentrations of PB (up to 100 μg/mL) and PBM. Additionally, PB and PBM did not significantly affect hemolysis rates, red blood cell morphology, or coagulation rates. These results suggest that Prussian blue-based materials, including PBM, are safe and exhibit excellent biocompatibility, making them conducive to further clinical trials and indicating no toxic side effects (Wu et al., 2023a).

#### 2.1.14 Glycyrrhetinic acid

Glycyrrhetinic acid (GA) is a bioactive compound derived from licorice root, a plant with a long history of medicinal use. The researchers developed a nanoplatform named GCMNPs (glycyrrhetinic acid-based nanoplatform) characterized by high specificity to cancer cells and diminished toxicity, aiming for effective cancer immunotherapy. GCMNPs, composed of glycyrrhetinic acid (GA) loaded into PLGA (poly (lactic-co-glycolic acid)) nanoparticles, were coated with leukocyte membrane vesicles for targeted delivery to cancer cells. GCMNPs, in combination with ferumoxytol, promoted a Fenton reaction, inducing ferroptosis in CRC cells. It was demonstrated that GCMNPs selectively targeted tumor cells, exhibited sustained-release properties, and induced ferroptosis by depleting GSH and downregulating GPX4. GA, known for its antitumor properties, interacts with the Fe/S center of the mitochondrial respiratory chain, leading to hydrogen peroxide production and subsequent oxidative stress. This interaction ultimately triggers the opening of transition pores and cell death pathways, including ferroptosis. Ferumoxytol, an iron-based therapy, catalytically disproportionate hydrogen peroxide to produce cytotoxic hydroxyl radicals through the Fenton reaction. These radicals induce lipid peroxidation, disrupt redox balance, and deplete glutathione levels, ultimately leading to ferroptotic cell death. Additionally, GA and ferumoxytol synergistically enhance ROS generation, further exacerbating oxidative stress. The downstream effects of these interactions include activation of stress response pathways, modulation of redox signaling, and initiation of immunogenic cell death processes. The combination of GCMNPs and ferumoxytol synergistically regulated cancer cell aggressiveness, reducing colony formation and migration. *In vivo* experiments using CRC and AML mouse models showed that GCMNPs, along with ferumoxytol and anti-PD-L1 treatment, resulted in significant tumor regression, reduced metastasis, and increased overall survival. The synergistic effect was attributed to increased ROS production, activation of CD8^+^ T cells, and enhanced lipid peroxidation, providing a promising strategy for cancer therapy. While glycyrrhetinic acid (GA) and ferumoxytol exhibit promising antitumor effects, their safety profiles warrant careful consideration, particularly regarding long-term or high-dose administration. GA, renowned for its therapeutic properties, has been linked to hepatotoxicity in some cases, limiting its clinical applicability. Therefore, monitoring liver function and optimizing dosages are imperative to mitigate potential adverse effects. Similarly, while ferumoxytol is FDA-approved for treating iron deficiency anemia, concerns exist regarding the risk of iron overload and oxidative stress, especially with high doses. Thus, cautious monitoring and dose management are essential to ensure the safety of these natural products in cancer therapy. In terms of safety, ferumoxytol may have an advantage over GA. However, the efficacy of each agent would ultimately depend on factors such as the specific cancer type, treatment context, and individual patient characteristics. Further clinical research is needed to directly compare the efficacy and safety of GA and ferumoxytol in cancer therapy (Li Q. et al., 2022).

## 3 Natural products targeting NRF2 in CRC

Nuclear factor erythroid 2-related factor 2 (NRF2) is crucial in regulating ferroptosis, as it serves as a master regulator of cellular antioxidant responses by controlling the expression of genes involved in redox homeostasis. Its actions include promoting the synthesis of the antioxidant GSH, regulating iron homeostasis through ferritin and ferroportin, and maintaining the activity of key enzymes like GPX4 that protect cells from lipid peroxidation. When activated, NRF2 translocates into the nucleus and binds to the antioxidant response element (ARE) in the promoters of target genes. GPX4 is an antioxidant enzyme crucial for protecting cells from lipid peroxidation and ferroptosis, while SLC7A11 is a component of the cystine/glutamate antiporter system Xc^−^, contributing to cellular antioxidant defense. The activation of NRF2 leads to the transcriptional upregulation of GPX4 and SLC7A11, among other genes, enhancing the cell’s ability to counteract oxidative stress and maintain redox balance, thereby promoting cell survival under stressful conditions. NRF2’s role in suppressing oxidative stress and lipid peroxidation makes it a key player in preventing ferroptosis. However, the relationship between NRF2 and ferroptosis can be context-dependent, highlighting the intricate interplay of cellular mechanisms in response to oxidative stress (Anandhan et al., 2020). In the initial phases of colon tumorigenesis, the activation of NRF2 provides a protective role by assisting in the elimination of carcinogens and suppressing inflammation. Nevertheless, in later stages, increased NRF2 expression in cancer cells is associated with unfavorable prognosis and resistance to chemotherapy. Utilizing phytochemical compounds sourced from natural origins emerges as a promising approach to target NRF2 in CRC. Flavonoids, such as epigallocatechin gallate (EGCG) found in green tea, sulforaphane derived from broccoli, and active components of garlic like allicin and S-allylmercapto-cysteine have exhibited the ability to activate NRF2. This activation contributes to chemopreventive effects by modulating processes like detoxification, antioxidation, and apoptosis (Zhu et al., 2020). These natural products can also induce ferroptosis in CRC through the regulation of NRF2 (Figure 1).

### 3.1 Lysionotin

Lysionotin, a flavonoid derived from Lysionotus pauciflorus Maxim, exhibits therapeutic potential in CRC by inducing ferroptosis. When applied to CRC cell lines like HCT116 and SW480, lysionotin sets off a series of events contributing to ferroptosis. Mechanistically, Lysionotin treatment leads to an increase in ROS levels and lipid peroxidation, accompanied by a decrease in GSH levels. This process is mediated by the downregulation of Nrf2 protein, a key regulator of cell redox homeostasis. Lysionotin-induced ferroptosis involves the inhibition of ferroptosis markers such as ferritin, GPX4, glutaminase, and xCT/SLC7A11, ultimately leading to CRC cell death. The induction of ferroptosis in CRC through lysionotin involves a complex modulation of NRF2 signaling, presenting a promising therapeutic approach for impeding CRC progression. Lysionotin may have a favorable safety profile for normal cells at certain concentrations. Nonetheless, further studies are necessary to comprehensively evaluate the safety of Lysionotin, especially in the context of long-term or high-dose administration, and its potential effects on various normal cell types (Gao et al., 2022).

### 3.2 Ginsenoside Rh3

Ginsenoside Rh3 (GRh3), a triterpene derived from ginsenoside Rg5, exhibits potent anticancer effects and is identified as a bacterial metabolite. Its efficacy in inhibiting CRC cell proliferation has been demonstrated both in laboratory settings and in live subjects. The underlying anticancer mechanism involves the initiation of pyroptosis and ferroptosis. GRh3 activates GSDMD-dependent pyroptosis while suppressing xCT/SLC7A11, thereby promoting ferroptosis through the Stat3/p53/NRF2 axis. GRh3 prevents NRF2 from entering the nucleus, reducing HO-1 expression, and consequently enhancing NLRP3 and caspase-1 expression. The activated caspase-1 then triggers GSDMD-dependent pyroptosis. Additionally, GRh3 induces a decrease in GSH levels and an accumulation of iron, lipid ROS, and MDA, which are characteristic of ferroptosis. The Stat3/p53/NRF2 axis emerges as a crucial regulatory pathway in GRh3-induced pyroptosis and ferroptosis in CRC cells. *In vivo* results corroborate these observations, highlighting the potential of GRh3 as a promising therapeutic agent for CRC by modulating dual cell death pathways, pyroptosis, and ferroptosis. The *in vitro* and *in vivo* studies demonstrating the efficacy of GRh3 in inhibiting CRC growth through the activation of pyroptosis and ferroptosis hold significant promise for potential clinical applications. Firstly, the ability of GRh3 to selectively induce cell death in CRC cells while sparing normal cells suggests its potential as a targeted therapy with reduced systemic toxicity compared to conventional chemotherapy. This could translate into fewer side effects and improved quality of life for patients undergoing treatment. Secondly, the mechanistic understanding of how GRh3 exerts its effects through the Stat3/p53/NRF2 axis provides insights into potential biomarkers for patient stratification and personalized treatment approaches. Furthermore, the demonstration of GRh3’s efficacy in inhibiting CRC growth in xenograft mouse models underscores its potential for further preclinical development and eventual clinical trials (Wu et al., 2023b).

### 3.3 Tagitinin C

Tagitinin C, a sesquiterpene lactone found in plants, has exhibited diverse pharmacological activities, including antitumor effects. Tagitinin C interacts with ferroptotic enzymes primarily through its activation of the Nrf2-HO-1 signaling pathway, mediated by ER stress-induced PERK activation. Upon treatment with tagitinin C, there is a rapid increase in ROS production, leading to oxidative stress within the cell. This oxidative stress triggers ER stress, activating PERK, which phosphorylates Nrf2. Phosphorylated Nrf2 translocates into the nucleus, where it upregulates the expression of downstream target genes, including HO-1. HO-1, in turn, catalyzes the degradation of heme into biliverdin, free iron, and carbon monoxide. The accumulation of free iron enhances lipid peroxidation and exacerbates oxidative damage, ultimately culminating in ferroptotic cell death. Additionally, tagitinin C-induced ER stress may further amplify the activation of the Nrf2-HO-1 pathway, contributing to the overall cytotoxic effect on cancer cells. This intricate interplay between tagitinin C, ferroptotic enzymes, and cellular pathways underscores its potential as a therapeutic agent for targeting colorectal cancer cells, particularly those resistant to conventional treatments. Further translational studies, including preclinical trials and clinical investigations, are warranted to validate the therapeutic potential of tagitinin C in colorectal cancer patients, with a focus on optimizing dosing regimens and assessing safety profiles. The demonstrated ability of tagitinin C to selectively induce ferroptotic cell death in colorectal cancer cells, including those resistant to traditional chemotherapeutic agents like erastin, suggests its potential as a novel therapeutic strategy for combating drug resistance in colorectal cancer patients. Moreover, the synergistic effect observed when tagitinin C is combined with erastin indicates the possibility of enhancing treatment efficacy through combination therapy (Wei et al., 2021). The safety profile of tagitinin C is an essential consideration for its potential clinical application. While tagitinin C has demonstrated promising anticancer effects in preclinical studies, including its ability to induce ferroptosis in colorectal cancer cells, its safety in humans remains to be fully elucidated. As with any potential therapeutic agent, thorough evaluation of tagitinin C’s toxicity profile, pharmacokinetics, and potential adverse effects is necessary before clinical use. Additionally, further research is needed to assess tagitinin C’s potential interactions with other medications and its long-term effects on vital organs and physiological systems. Preclinical studies, including animal toxicology studies, can provide valuable insights into tagitinin C’s safety profile, guiding the design of subsequent clinical trials.

## 4 Natural products targeting lipid metabolism in CRC

Lipid metabolism is intricately linked to ferroptosis. Arachidonate lipoxygenases (ALOX), particularly 15-lipoxygenase (15-LOX), initiate ferroptosis by oxidizing polyunsaturated fatty acids (PUFAs) like arachidonic acid (AA) and adrenic acid (AdA). Concurrently, acyl-CoA synthetase long-chain family members (ACSLs), notably ACSL4, play a crucial role by incorporating PUFAs into cellular membranes, making cells more susceptible to lipid peroxidation. This interplay leads to the generation of lipid hydroperoxides, ultimately triggering ferroptosis. In addition to the roles of ACSL4 and ALOX15 in lipid metabolism and ferroptosis, several other key regulatory factors play significant roles. Phospholipase D2 (PLD2) generates phosphatidic acid, which is pivotal in signaling pathways that affect cell membrane composition and integrity, influencing ferroptosis sensitivity (Park et al., 2023). Acyl-CoA synthetase long-chain family member 3 (ACSL3) also plays a crucial role by facilitating the incorporation of polyunsaturated fatty acids into phospholipids, thus making membranes susceptible to peroxidation (Klasson et al., 2022). Similarly, calcium-independent phospholipase A2 beta (iPLA2β) is involved in the remodeling of membrane phospholipids by releasing oxidized fatty acids, a step critical for the execution of ferroptosis (Chen et al., 2021). 1-acylglycerol-3-phosphate O-acyltransferase 3 (AGPAT3) is involved in the biosynthesis of phospholipids, and its activity affects the lipid composition of cellular membranes, thereby impacting ferroptosis (Zou et al., 2020). PLA2G6 (iPLA2β) plays a crucial role in ferroptosis by hydrolyzing peroxidized phospholipids, particularly Hp-PEs, which are central to the propagation of lipid peroxidation. The absence of PLA2G6 leads to increased accumulation of peroxidized lipids, heightening susceptibility to ferroptosis. This enzyme’s activity is critical in controlling lipid peroxidation and modulating cell vulnerability to ferroptotic cell death, highlighting its importance in cellular protection mechanisms against ferroptosis (Lee et al., 2021). Collectively, these proteins contribute to the complex regulation of lipid metabolism, underscoring their potential as targets for modulating ferroptosis in therapeutic strategies, particularly in cancer treatment where altering lipid peroxidation can influence disease progression. Understanding these regulatory mechanisms provides insights into potential therapeutic strategies, particularly in the context of diseases like cancer where ferroptosis dysregulation is common (Figure 1) (Li and Li, 2020).

### 4.1 ACSL4

Bromelain, derived from pineapple stem, demonstrates anti-cancer effects in CRC, particularly in cells with K-Ras mutations. Induction of ferroptosis is achieved by down-regulating ACSL4, a gene involved in lipid metabolism in CRC. K-Ras mutant CRC cells, expressing higher levels of ACSL4 mRNA, are more sensitive to erastin-induced ferroptosis. Bromelain treatment enhances erastin-induced cell death in K-Ras mutant cells, possibly by further disrupting lipid metabolism. The process involves an increase in ROS accumulation, a hallmark of ferroptosis. Thus, bromelain’s induction of ferroptosis in CRC cells with K-Ras mutations is associated with the modulation of lipid metabolism, suggesting a potential therapeutic avenue for K-Ras mutant CRC. The *in vitro* studies demonstrating the efficacy of bromelain in inhibiting the proliferation of Kras mutant CRC cells and inducing ferroptosis have significant implications for potential clinical applications. Firstly, the specific targeting of Kras mutant CRC cells by bromelain suggests its potential as a targeted therapy for patients with Kras mutant CRC, who often exhibit poorer prognosis and limited treatment options. Secondly, the induction of ferroptosis by bromelain in CRC cells offers a novel therapeutic approach for CRC treatment, particularly for patients with resistant or refractory disease. Additionally, the ability of bromelain to modulate the expression of genes and miRNAs involved in CRC progression further underscores its potential as a multifaceted therapeutic agent. Moreover, the findings from *in vivo* studies, demonstrating the efficacy of bromelain in reducing intestinal inflammation and polyp formation in a mouse model of CRC with Kras mutation, provide strong preclinical evidence for its therapeutic potential (Park et al., 2018). However, further investigation is necessary to assess the potential adverse effects of bromelain on normal cells.

### 4.2 ALOX15

Docosahexaenoic acid (DHA), an omega-3 polyunsaturated fatty acid, is being investigated for its ability to trigger ferroptosis in CRC. This essential fatty acid, commonly found in fish and fish oil supplements, plays a crucial role in various physiological processes. DHA interacts with ferroptotic enzymes and influences cellular pathways involved in ferroptosis. Firstly, DHA enhances ferroptosis by promoting lipid peroxidation through its susceptibility to oxidation, leading to increased levels of ROS and lipid peroxides. This effect is mediated by both ALOX5-dependent and independent pathways, where ALOX5 catalyzes the conversion of DHA to peroxides, contributing to ROS generation. Additionally, DHA augments ferroptotic cell death independently of apoptosis or autophagy pathways, indicating a distinct regulatory mechanism. Furthermore, DHA-mediated ferroptosis is associated with altered fatty acid composition in cell membranes, characterized by increased levels of highly unsaturated phospholipids. These findings suggest that DHA plays a pivotal role in sensitizing cells to ferroptosis by modulating lipid metabolism and oxidative stress pathways, ultimately leading to cell death. *In vitro* findings suggest that combining DHA with ferroptosis inducers could potentiate cancer therapy by selectively targeting tumor cells. Moreover, *in vivo* studies show that dietary supplementation with DHA-containing fish oil increases DHA levels in tumor tissue and enhances the efficacy of ferroptosis-based treatment in reducing tumor size and weight. These results indicate the potential clinical utility of incorporating DHA supplementation into cancer treatment regimens to improve therapeutic outcomes (Shan et al., 2022).

## 5 Natural products targeting iron metabolism in CRC

**FIGURE 2:**
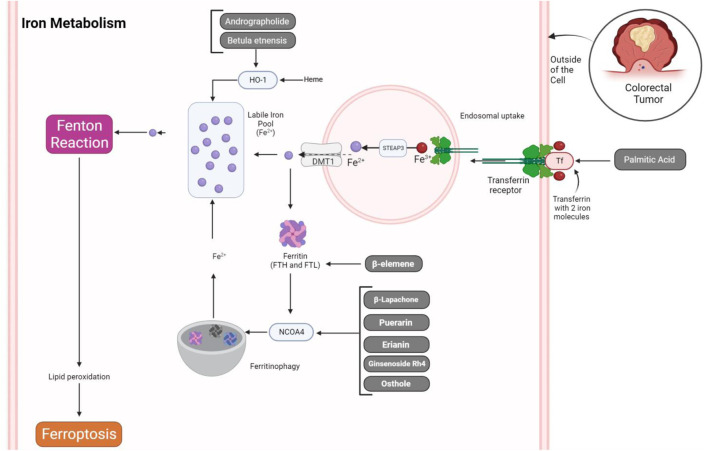
Natural products regulating iron metabolism in ferroptosis of CRC cells. The process of iron uptake by transferrin and its receptor leads to their endosomal uptake. Within the endosome, the conversion of Fe^3+^ to Fe^2+^ is facilitated by STEAP3, followed by exportation through DMT1. This exported iron then combines with the labile iron pool, contributing ions for the Fenton reaction and inducing ferroptosis via lipid peroxidation. Conversely, iron ions can be stored within ferritin molecules or undergo degradation by NCOA4, thereby promoting the generation of free iron through the ferritinophagy process. Natural products can influence these regulatory mechanisms, potentially accelerating ferroptosis in CRC cells.

Iron metabolism plays a central role in the process of ferroptosis. Key evidence supporting the significance of iron in ferroptosis includes the inhibitory effect of iron chelators on cell death and the observation of elevated labile iron levels during ferroptosis induction. Additionally, the supplementation of exogenous iron intensifies cell sensitivity to ferroptosis triggers, and an excess of heme and non-heme iron can directly initiate ferroptosis. Iron-containing enzymes, such as NOXs and CYP, are implicated in lipid peroxidation, a hallmark of ferroptosis, and iron-mediated ROS production via the Fenton reaction further promotes lipid peroxidation in this context. Various regulators of iron metabolism, such as transferrin and lactotransferrin, transferrin receptors, SLC39 family members, and ferritin, intricately modulate ferroptosis. This complex interplay highlights potential therapeutic targets for conditions associated with aberrant cell death processes (Figure 2) (Chen X. et al., 2020).

### 5.1 Transferrin

In ferroptosis, transferrin assumes a pivotal role as a vital regulator of iron accessibility. This glycoprotein binds to iron in the bloodstream and facilitates its transport to various tissues. Specifically, transferrin plays a crucial part in cell death induced by amino acid starvation. During this process, the presence of transferrin in serum is essential for triggering ferroptosis (Feng et al., 2020). The transferrin receptor (TFRC) is vital for cellular iron uptake, and its overexpression in CRC has been explored in a study. The research unveils that APC gene loss-driven β-catenin activation induces TFRC in CRC. TFRC, facilitating intratumoral iron accumulation, boosts tankyrase activity, thereby contributing to β-catenin signaling. Disrupting TFRC reduces colonic iron levels, impairs tankyrase activity, and stabilizes AXIN2, resulting in the suppression of the β-catenin/c-Myc/E2F Transcription Factor 1/DNA polymerase delta1 (POLD1) axis. POLD1 deficiency, TFRC disruption, and iron chelation induce DNA replication stress, activate DNA damage responses, trigger apoptosis, and reduce colon tumor growth. Combining iron chelators with DNA-damaging agents enhances DNA damage responses and decreases colon tumor cell growth (Kim et al., 2023). Therefore, targeting transferrin or its receptor can be considered as a novel strategy to hinder tumorigenesis in CRC.

#### 5.1.1 Palmitic acid

Kuang et al. explored the impact of palmitic acid (PA), a prevalent saturated fatty acid present in palm oil and certain animal products, on colon cancer cells. Their investigation revealed that PA induces ferroptosis, characterized by non-apoptotic and non-necrotic processes. The underlying mechanism involves PA triggering the release of calcium through endoplasmic reticulum (ER) stress, resulting in disruptions in transferrin (TF) transport and imbalances in iron ions. The sensitivity of colon cancer cells to PA-induced ferroptosis is linked to the expression of CD36. Excessive iron, a crucial factor in regulating ferroptosis, is shown to be a consequence of the heightened internalization of the iron-TF-TF receptor complex. The study underscores the interconnected roles of calcium and iron in PA-induced ferroptosis and emphasizes the clinical relevance of CD36, noting elevated expression in colon adenocarcinoma tissues, which correlates with a poorer prognosis in specific patients. *In vivo* experiments utilizing xenograft models validate the anti-tumor effectiveness of PA and underscore the pivotal role of CD36 in the ferroptotic response. PA selectively inhibits the proliferation and induces cell death in certain colon carcinoma cells, namely HT29 and HCT116, while not significantly affecting the proliferation or death rates of NCM460 normal human colon cells. This selective effect indicates that PA may have a preferential cytotoxic action against cancer cells compared to normal cells (Kuang et al., 2023).

### 5.2 Ferritin

Ferritin plays a crucial role in maintaining cellular iron balance and regulating ferroptosis. As an iron storage protein, ferritin prevents the buildup of free iron in the cytoplasm, protecting cells from oxidative stress and reactive oxygen species generated through the Fenton reaction. Its controlled regulation, responsive to cellular iron levels, enables the safe storage and release of iron. By inhibiting ferroptosis and aiding in the secure storage of excess iron, ferritin contributes to cellular resistance against oxidative damage. The coordinated interactions among ferritin, iron storage processes, and iron release mechanisms are essential for preserving redox equilibrium and preventing disorders associated with imbalanced iron metabolism and ferroptosis (Chen X. et al., 2020; Qin et al., 2021).

#### 5.2.1 β-elemene

The compound β-elemene, derived from the Chinese herb Curcumae Rhizoma, exhibits strong anticancer effects in various cancers, including CRC. Despite its known benefits, its influence on ferroptosis and the responsiveness of KRAS mutant CRC cells to cetuximab treatment has not been investigated until now. Chen et al. unveiled a novel finding, demonstrating that the combined use of β-elemene and cetuximab exerts iron-induced cell death in CRC cells with KRAS mutations, resulting in decreased cell viability and increased iron-dependent lipid peroxidation. This combined treatment not only hampers cell proliferation, causing G0/G1-phase arrest but also enhances the vulnerability of KRAS mutant CRC cells to the treatment. Through its action, β-elemene induces ferroptosis, a form of iron-dependent cell death characterized by lipid peroxidation accumulation. This induction involves modulation of several key ferroptotic enzymes and proteins, including HO-1, transferrin, GPX4, SLC7A11, FTH1, and glutaminase. β-elemene treatment leads to increased ROS accumulation, depletion of GSH, and enhanced lipid peroxidation, ultimately culminating in ferroptotic cell death. Moreover, β-elemene exhibits inhibitory effects on EMT markers, such as Vimentin, N-cadherin, Slug, Snail, and MMP-9, while promoting the expression of the epithelial marker E-cadherin. These dual actions of β-elemene not only trigger ferroptosis but also inhibit EMT, collectively contributing to the suppression of cell proliferation, migration, and tumor growth in KRAS mutant CRC cells. The findings suggest these anti-cancer effects translate into promising therapeutic strategies for CRC patients with RAS mutations, who often exhibit resistance to conventional treatments. The observed suppression of tumor growth and metastasis in orthotopic murine colon cancer models further underscores the potential clinical relevance of β-elemene-based therapies. Given its natural origin and minimal toxicity demonstrated *in vivo*, β-elemene holds promise as a novel adjunctive therapy or combination regimen for improving treatment outcomes in KRAS mutant CRC patients, warranting further clinical investigations to validate its efficacy and safety (Chen P. et al., 2020).

### 5.3 HO-1

Heme oxygenase-1 (HO-1) plays a crucial role in protecting cells from ferroptosis. HO-1 functions by breaking down heme into biliverdin, carbon monoxide, and free iron. Biliverdin and its subsequent conversion to bilirubin have antioxidant properties, while carbon monoxide has anti-inflammatory effects. The release of free iron during this process may trigger ferritin synthesis, sequester iron and prevent its participation in ferroptotic reactions. Overall, HO-1 exerts a cytoprotective role in ferroptosis by modulating oxidative stress, inflammation, and iron homeostasis (Chiang et al., 2018). However, it is essential to recognize that excessive activation of HO-1 can have adverse effects on cellular function. Overexpression or prolonged activation may lead to the accumulation of toxic byproducts, disrupt cellular heme balance, and contribute to disease pathogenesis in conditions such as cancer and neurodegenerative diseases. Therefore, while HO-1 serves as a protective mechanism against oxidative stress and inflammation, maintaining a balance in its activation is crucial to prevent potential cellular toxicity and pathological consequences (Chiang et al., 2018). Targeting HO-1 with natural products is an emerging strategy in CRC therapy. Natural compounds have demonstrated the ability to modulate HO-1 expression, potentially sensitizing CRC cells to ferroptosis and other forms of cell death.

#### 5.3.1 Andrographolide

Andrographolide, a natural compound found in Andrographis paniculata, possesses various biological activities, including anti-cancer properties. Transcriptomic profiling and validation assays revealed upregulation of key ferroptosis-related genes, including HMOX1, GCLC, and GCLM, following andrographis treatment. These genes are critical mediators of ferroptosis induction, suggesting that andrographis promotes ferroptotic cell death in CRC. Additionally, andrographis treatment resulted in increased protein expression of HMOX1 and GCLM, further supporting its role in ferroptosis induction. The activation of ferroptosis by andrographis is associated with its chemosensitizing effects, as evidenced by enhanced cytotoxicity and apoptosis in chemoresistant CRC cells. Furthermore, andrographis-mediated inhibition of the β-catenin/Wnt-signaling pathway was observed, indicating a dual mechanism of action in CRC treatment. These findings underscore the potential of andrographis as a novel therapeutic agent for CRC, offering insights into its molecular mechanisms and therapeutic efficacy. By overcoming chemoresistance and enhancing the efficacy of conventional chemotherapy, andrographolide offers a novel approach to improving patient outcomes. Furthermore, its safety profile and multitargeted effects make it an attractive candidate for combination therapies, potentially reducing the dosage and toxicity of conventional chemotherapeutic drugs. Clinical trials evaluating the efficacy and safety of andrographolide in CRC patients could pave the way for its incorporation into standard treatment regimens, providing a more effective and well-tolerated (Sharma et al., 2020).

#### 5.3.2 Betula etnensis Raf

The methanolic extract of Betula etnensis Raf. bark has been investigated for its effects on colorectal cancer cells, particularly focusing on the induction of HO-1. The methanolic extract of Betula etnensis Raf. bark demonstrates significant interactions with ferroptotic proteins and regulators, leading to distinct downstream effects on cellular pathways. Through MTT bioassays, LDH release measurements, annexin-V/7-AAD assays, ROS and LOOH level evaluations, and total thiol group assessments, it was observed that the extract induces dose-dependent reductions in cell viability, increases in LDH release, and elevations in ROS and LOOH levels, while decreasing total thiol group levels. Notably, the extract induces necrosis-mediated antiproliferative effects, as evidenced by annexin-V/7-AAD results. Additionally, the extract leads to a significant increase in HO-1 protein expression, indicating its involvement in ferroptosis induction. Furthermore, cell cycle analysis reveals a dose-dependent cell cycle arrest at G0/G1 and S phases, accompanied by a decrease in the G2/M phase. These findings collectively suggest that the methanolic extract of Betula etnensis Raf. bark exerts its cytotoxic effects through the induction of ferroptosis, mediated by alterations in cellular redox balance and modulation of key regulatory proteins such as HO-1, ultimately resulting in cell cycle arrest and necrosis-mediated cell death. Such promising *in vitro* results indicate the potential clinical applications of Betula etnensis Raf. bark extract as a novel therapeutic agent for colon cancer treatment. Further preclinical and clinical studies are warranted to validate its efficacy and safety profiles, paving the way for its development as a complementary or alternative therapy for colorectal cancer patients. While these findings suggest potential therapeutic benefits against cancer, they also raise concerns about potential toxicities associated with the extract. The observed increase in necrotic cell death and oxidative stress markers such as ROS and lipid peroxidation may indicate cellular damage beyond cancer cells. However, further studies are needed to comprehensively assess the safety profile of the extract, including acute and chronic toxicity evaluations, as well as investigations into potential adverse effects on normal cells and tissues. Additionally, determining the maximum tolerated dose and examining the extract’s effects in animal models would provide valuable insights into its safety and toxicity profiles (Malfa et al., 2019).

### 5.4 NCOA4

NCOA4 plays a crucial role in ferritinophagy, a specialized form of autophagy responsible for breaking down ferritin, the primary cellular iron storage protein. Identified as a significantly enriched protein in autophagosomes, NCOA4 directly engages with ferritin subunits, facilitating the transport of iron-loaded ferritin to lysosomes for degradation. Depleting NCOA4 hinders ferritin turnover, leading to decreased cellular iron availability. NCOA4 levels are finely controlled by cellular iron levels, with high iron levels causing NCOA4 degradation and reduced ferritinophagy, while low iron levels result in elevated NCOA4, promoting ferritinophagy to release iron for cellular use. This regulatory mechanism ensures a balanced iron storage and release, underscoring NCOA4’s central role in preserving cellular iron homeostasis through ferritinophagy (Quiles del Rey and Mancias, 2019). The expression of NCOA4 is positively correlated with survival time of patients with colon adenocarcinoma (Gu et al., 2022). Herein, we introduce some phytochemicals that can target NCOA4 in CRC.

#### 5.4.1 β-Lapachone

β-Lapachone, a natural compound from the lapacho tree, effectively combats CRC by inducing ferroptosis. This occurs through β-Lapachone’s activation of NQO1, causing an increase in intracellular ROS and subsequent lipid peroxidation, initiating ferroptosis. β-Lapachone interacts with ferroptotic enzymes primarily by inducing intracellular iron overload and ROS generation in CRC cells. This leads to the downregulation of key ferroptosis regulators such as SLC7A11 and GPX4, resulting in lipid peroxidation and ferroptotic cell death. Additionally, β-Lapachone activates the JNK signaling pathway, further promoting ferroptosis and ferritinophagy. Mechanistically, β-Lapachone triggers ferritinophagy via upregulation of NCOA4 and downregulation of FTH1, leading to the release of free iron ions and subsequent lipid peroxidation. Moreover, β-Lapachone-induced activation of the JNK pathway enhances ferroptosis and ferritinophagy, possibly through the modulation of autophagy-related genes. These findings elucidate the intricate molecular mechanisms by which β-Lapachone induces ferroptosis and ferritinophagy in CRC cells, offering potential therapeutic avenues for cancer treatment targeting ferroptosis. These findings provide valuable insights into the mechanisms underlying β-Lapachone’s therapeutic potential against CRC. Translating these results to potential clinical applications, β-Lapachone could serve as a novel therapeutic agent for CRC treatment, particularly for patients with resistant or refractory tumors. Further clinical studies are warranted to validate the efficacy and safety of β-Lapachone in CRC patients and to explore its potential synergistic effects with existing cancer therapies (Zhao et al., 2024). However, the safety characteristics of β-Lapachone have not been thoroughly examined and require additional research to understand any possible adverse effects.

#### 5.4.2 Puerarin

The natural isoflavone puerarin, derived from the root of Pueraria lobata, shows promise in inhibiting CRC cell growth by modulating key proteins, upregulating Bax, and downregulating c-Myc and Bcl-2. Puerarin treatment led to a dose-dependent reduction in cell viability, accompanied by decreased glutathione levels and increased ROS and MDA levels. Downregulation of GPX4 and the reversal of puerarin effects by ferroptosis inhibitors suggest puerarin induces ferroptosis in CRC cells. Additionally, puerarin induced autophagy, evidenced by LC3-II upregulation, and inhibiting autophagy reversed these effects. The pivotal role of autophagy, particularly NCOA4-mediated ferritin degradation, in puerarin-induced ferroptosis in CRC cells has been highlighted. Knocking down NCOA4 reversed the observed effects, indicating that puerarin triggers autophagy-dependent ferritinophagy through NCOA4, ultimately leading to ferroptosis in CRC cells (Lian et al., 2023). Yet, the safety profile and *in vivo* effects of puerarin have not been extensively studied and requires further investigation.

#### 5.4.3 Ginsenoside Rh4

Ginsenoside Rh4, a natural compound derived from ginseng, has shown significant inhibitory effects on CRC. Ginsenoside Rh4 interacts with ferroptotic proteins in CRC cells by inducing alterations in their expression levels and activities. Specifically, Rh4 leads to the upregulation of proteins promoting ferroptosis, such as KEAP1, NCOA4, and DMT1/SLC11A2, while downregulating proteins inhibiting ferroptosis like NRF2, FTH1, xCT/SLC7A11, and GPX4. These changes result in increased cellular accumulation of iron, lipid ROS, and MDA, as well as decreased concentrations of GSH, indicative of ferroptosis induction. Moreover, Rh4-induced ferroptosis is closely linked to the activation of the autophagy pathway, as inhibition of autophagy reverses Rh4-induced ferroptosis. This interplay between autophagy and ferroptosis contributes to the anti-cancer effects of Rh4 by promoting cell death and inhibiting CRC cell proliferation. Additionally, Rh4 treatment activates the p53 signaling pathway and ubiquitin-mediated proteolysis, further enhancing its inhibitory effects on CRC growth both *in vitro* and *in vivo*. The promising results of Ginsenoside Rh4 in both *in vitro* and *in vivo* studies hold significant potential for clinical applications in CRC treatment. *In vitro* experiments demonstrated Rh4’s ability to inhibit CRC cell proliferation by activating autophagy and inducing ferroptosis, mechanisms that were further validated *in vivo* in xenograft mouse models. These findings suggest Rh4 is a potential therapeutic agent for CRC, capable of suppressing tumor growth without causing significant damage to the organism. Given these encouraging results, further preclinical studies and clinical trials could explore the therapeutic efficacy and safety of Rh4 in CRC patients, potentially leading to the development of novel treatment strategies for this prevalent malignancy (Wu et al., 2022).

## 6 Natural products targeting AMP/mTOR in CRC

Adenosine monophosphate-activated protein kinase (AMPK) assumes a crucial role in regulating ferroptosis, triggered by fluctuations in the intracellular ADP/ATP ratio. Activation of AMPK initiates downstream changes that intensify ferroptosis, including facilitating ATP breakdown, hindering anabolic processes, and heightening susceptibility to GPX4-dependent ferroptosis. Moreover, activated AMPK induces autophagy, selectively breaking down factors opposing ferroptosis and ferritin, impacting iron metabolism and fostering lipid peroxide buildup. AMPK also maintains cellular amino acid equilibrium by controlling mTORC1, influencing ferroptosis. Significantly, AMPK suppresses system Xc^−^, leading to diminished cystine uptake, compromised glutathione synthesis, and reduced GPX4 function, fostering an environment conducive to ferroptotic cell demise. Additionally, in the context of ferroptosis induction, the inhibitory impact of AMPK on mTORC1 is crucial, as mTORC1 inhibition correlates with decreased SREBP1 activity, reducing SCD1 expression and enhancing lipid ROS accumulation (Wang X. et al., 2023). Herein, we introduce several naturally derived compounds that can trigger ferroptosis in CRC cells via ferroptosis.

### 6.1 AMPK

#### 6.1.1 Gambogenic acid

Gambogenic acid (GNA), a bioactive compound found in Gamboge, demonstrates potential anti-tumor effects in CRC by triggering ferroptosis. GNA activates AMPKα, a cellular energy sensor, in CRC cells. Although AMPKα activation is linked to ferroptosis, GNA-induced ferroptosis in CRC is only partly reliant on AMPKα signaling. Instead, GNA alters iron metabolism, leading to increased levels of transferrin and ferritin, and disrupts the SLC7A11/GPX4 axis. Specifically, GNA enhances SLC7A11, a component of the cystine/glutamate antiporter system Xc−, while suppressing GPX4 expression, a crucial enzyme in the cellular antioxidant defense system. This dual effect promotes lipid peroxidation and the accumulation of lipid hydroperoxides, ultimately inducing ferroptosis in CRC cells. The *in vitro* and *in vivo* results demonstrate the efficacy of GNA in inhibiting CRC proliferation and inducing ferroptosis, which hold promising implications for potential clinical applications. Firstly, GNA’s ability to inhibit CRC cell viability and colony formation *in vitro* suggests its potential as a standalone therapeutic agent or as part of combination therapy regimens for CRC treatment. Additionally, the observed inhibition of tumor growth in mouse xenograft models further supports GNA’s efficacy *in vivo*, indicating its potential for translational application in human patients. Furthermore, GNA’s mechanism of action, particularly its induction of ferroptosis through modulation of iron metabolism and antioxidant defense pathways, presents a novel therapeutic approach for CRC management. Given the limited treatment options and therapeutic resistance often observed in CRC, GNA’s ability to target multiple pathways involved in cancer progression offers a promising avenue for the development of targeted therapies. Specifically, the results indicate that GNA treatment had no significant effect on the body weight of mice compared to the control group. This finding suggests that GNA has little toxicity on the mice used in the experiment. However, it’s essential to note that the assessment of toxicity in animal models may not directly translate to human toxicity. Further studies, including comprehensive toxicity assessments and pharmacokinetic analyses, would be necessary to fully evaluate the safety profile of GNA for potential clinical applications in humans (Ma et al., 2023).

#### 6.1.2 Butyrate

Sodium butyrate (NaB) exerts its effects by influencing key enzymes and pathways involved in cellular regulation. NaB treatment leads to an increase in ROS production, particularly lipid ROS, contributing to ferroptotic cell death. Mechanistically, NaB downregulates the expression of critical ferroptotic enzymes and regulators, such as SLC7A11 and NRF2, via the GSK3β-β-TRCP1-NRF2 pathway. This downregulation of SLC7A11, a cystine transporter crucial for glutathione synthesis, reduces cellular antioxidant capacity, rendering cells more susceptible to lipid peroxidation and subsequent ferroptosis. Furthermore, NaB inhibits mTORC1 activation through cAMP-PKA signaling, leading to decreased expression of GPX4, a key regulator of ferroptosis. Additionally, NaB-induced AKT inhibition activates GSK3β, further promoting NRF2 degradation and ferroptosis induction. The “butyrate paradox” illustrates how butyrate, a short-chain fatty acid, has contrasting effects on normal versus cancerous colon cells. In normal cells, butyrate promotes growth by activating histone acetyltransferase 1 (HAT1), enhancing gene expression essential for cell maintenance. In colon cancer cells, however, butyrate inhibits histone deacetylase (HDAC), leading to increased histone acetylation, which suppresses growth and triggers apoptosis. This differential action of butyrate suggests its potential as a targeted therapy that can selectively affect cancer cells while supporting the vitality of normal cells. *In vitro* and *in vivo* studies reveal that butyrate’s dual role as an HDAC inhibitor in cancer cells and an activator of HAT1 in normal cells offers a promising basis for clinical applications, particularly in treating colon cancer. These studies elucidate the potential of butyrate to induce apoptosis in cancer cells while preserving healthy cells, supported by experiments on cultured cells and animal models that confirm its therapeutic mechanisms and overall safety. The insights gained could lead to butyrate being developed as a component of combination therapies for colon cancer, potentially extending to other cancers and metabolic diseases where butyrate’s modulation of gene expression plays a beneficial role (Wang G. et al., 2023).

### 6.2 mTOR

#### 6.2.1 Curcumin

Curcumin exerts its effects on cellular pathways primarily through its interactions with targeted proteins involved in redox homeostasis and cell survival. By downregulating key antioxidant proteins such as GPX4 and SLC7A11, curcumin disrupts the cellular defense against oxidative stress. This disruption leads to an accumulation of ROS and lipid peroxides, triggering ferroptosis. Moreover, curcumin suppresses the PI3K/Akt/mTOR signaling pathway, which is known to inhibit ferroptosis and promote cell survival. Through its inhibition of phosphorylated forms of PI3K, Akt, and mTOR, curcumin enhances the susceptibility of CRC cells to ferroptotic cell death. These dual mechanisms of action, targeting both antioxidant enzymes and pro-survival signaling pathways, contribute to curcumin’s selective induction of ferroptosis in CRC cells, thereby inhibiting their proliferation and offering potential therapeutic benefits for CRC treatment (Chen M. et al., 2023). Curcumin’s effects on normal cells appear to be less pronounced compared to its effects on cancer cells. Studies have shown that curcumin selectively induces ferroptosis in cancer cells while sparing normal cells. This selectivity may be attributed to differences in the redox status and vulnerability to oxidative stress between cancerous and normal cells. Normal cells typically possess robust antioxidant defense mechanisms that protect them from oxidative damage, whereas cancer cells often exhibit increased susceptibility to oxidative stress due to their altered metabolism and heightened proliferative state. Therefore, curcumin’s modulation of redox homeostasis and induction of ferroptosis may have minimal impact on normal cells, allowing them to maintain their viability and function. Additionally, curcumin has been reported to possess antioxidant and anti-inflammatory properties, which could potentially benefit normal cells by protecting them from oxidative damage and inflammation (Costantino et al., 2022). Despite its promising therapeutic properties, curcumin faces limitations in cancer treatment due to its low solubility in water and poor bioavailability. Clinical studies administering curcumin orally at high doses have revealed rapid biotransformation, leading to minimal levels of free curcumin in the bloodstream. To overcome these obstacles, various approaches have been explored to improve curcumin’s effectiveness and absorption. These include chemical modifications and the development of curcumin analogs to enhance stability and solubility. Additionally, nanoformulations like liposomal curcumin, protein nanoparticles, and exosomal curcumin have shown improved bioavailability and anti-cancer effects in both preclinical and clinical trials. Furthermore, novel delivery systems such as BCM-95^®^ CG have significantly increased curcumin’s relative bioavailability compared to conventional formulations. However, challenges remain in translating curcumin’s potential into clinical practice, including its narrow therapeutic window, inefficient absorption, and rapid metabolism. Although clinical trials investigating curcumin formulations like curcumin phytosome and nanostructured lipid curcumin particles have shown promise in managing colorectal cancer, further research is necessary to overcome these hurdles and establish curcumin as an effective cancer treatment option (Ojo et al., 2022).

### 6.3 AMPK/mTOR

#### 6.3.1 Osthole

Osthole, a natural coumarin derived from Cnidium spp. and other Apiaceous plants, exhibits a diverse range of pharmacological effects, including anti-inflammatory, neuroprotective, and anticancer properties. In CRC, particularly in KRAS-mutant cells, osthole demonstrates potent inhibitory effects on malignant phenotypes. Mechanistically, osthole induces ferroptosis, a form of programmed cell death characterized by iron-dependent lipid peroxidation. Osthole treatment leads to the generation of ROS and depletion of GSH, along with an increase in cellular iron levels. These alterations trigger lipid peroxidation and ultimately cell death. Importantly, osthole inhibits autophagy but requires lysosomal activity for its ferroptotic effects. Furthermore, osthole suppresses AMPK/Akt/mTOR signaling, sensitizing cells to ferroptosis induction. This multifaceted approach culminates in the inhibition of cancer cell proliferation and tumor growth, suggesting osthole as a promising candidate for combination therapy, especially when used alongside existing drugs, like cetuximab, in the treatment of KRAS-mutant CRC. These mechanisms highlight osthole’s potential as a therapeutic agent for CRC, particularly in KRAS-mutant cases where conventional treatments often fall short. Additionally, osthole enhances the cytotoxicity of cetuximab, suggesting a promising combination therapy strategy. These findings pave the way for further clinical investigations to explore osthole’s efficacy and safety in CRC treatment, potentially offering new avenues for improving patient outcomes in the future. The evidence suggests that osthole exhibits lower toxicity towards normal cells compared to cancer cells, indicating its potential as a selective therapeutic agent. This selectivity is particularly advantageous in cancer treatment, where minimizing harm to healthy tissues is crucial. Osthole’s ability to preferentially target cancer cells may be attributed to various factors, including differences in cellular metabolism and susceptibility to specific mechanisms of cell death induced by osthole, such as ferroptosis (Farooq et al., 2019; Zhou et al., 2023).

## 7 Natural products targeting other ferroptosis regulators in CRC

In comparison to the well-established regulators governing iron metabolism, amino acids, and lipid metabolism, ferroptosis regulators exhibit a remarkable diversity. Notably, recent research has unveiled novel players in this intricate network, notably p53 and ferroptosis suppressor protein 1 (FSP1). These emerging regulators have garnered attention for their potential implications in the context of colorectal cancer. Investigations into ferroptosis and its regulators have expanded beyond conventional boundaries, exploring the therapeutic potential of natural products in modulating these newly identified targets.

### 7.1 FSP1

FSP, plays a crucial role in suppressing ferroptosis. Instead of relying on the traditional glutathione pathway, FSP1 utilizes the FSP1-CoQ10-NAD(P)H axis and Coenzyme Q10 as an antioxidant to prevent lipid peroxidation. It is involved in a unique vitamin K oxidation-reduction cycle, acting as a vitamin K reductase to inhibit lipid peroxidation. FSP1 contributes to the ESCRT III-dependent membrane repair pathway, hindering the progression of ferroptosis. The regulation of FSP1 involves factors like NRF2, p53, and METTL3 at transcriptional and post-transcriptional levels. FSP1 undergoes myristoylation for membrane localization, and its clinical implications extend to various conditions such as cancer, neurodegenerative disorders, and organ damage. Targeting FSP1 emerges as a promising therapeutic strategy for diseases associated with ferroptosis, and ongoing research explores specific inhibitors for FSP1 (Li et al., 2023). In nonmetastatic colorectal cancer, the expression of FSP-1 in cancer cells is associated with adverse long-term oncological outcomes. Patients with FSP-1 expression demonstrate reduced 10-year overall survival (OS) and an increased risk of systemic recurrence (SR) (Im et al., 2022). In KRAS-mutated cells, oncogenic KRAS induces resistance to ferroptosis. This resistance is mediated by the upregulation of FSP1 through activation of the NRF2 and MAPK pathways. FSP1, an NADH ubiquinone oxidoreductase, protects cells from ferroptosis by preventing lipid peroxidation. Thus, FSP1 plays a crucial role in promoting cellular transformation and tumor initiation in KRAS-mutated cells, providing a survival advantage during early stages of tumorigenesis. Clinical relevance is supported by elevated FSP1 expression in human KRAS-driven cancers, correlating with poor outcomes. Targeting FSP1, in combination with other therapeutic approaches, may present a potential strategy for treating KRAS-mutated cancers like colon cancers (Müller et al., 2023).

#### 7.1.1 Curcumin and Andrographis

Curcumin and andrographis both promote ferroptosis in colorectal cancer through distinct molecular pathways involving key proteins like GPX4 and FSP1. Curcumin inhibits GPX4, an enzyme critical in preventing lipid peroxidation, and potentially reduces the activity or expression of FSP1, which supports lipid repair. This results in increased lipid peroxidation and cell death. Andrographis, meanwhile, affects ferroptosis by reducing cystine uptake via the system Xc^−^ transporter, indirectly decreasing glutathione levels and thereby inhibiting GPX4 activity. Yet, these effects should be investigated in animal and clinical studies. In addition, potential side effects should be explored in the future (Miyazaki et al., 2023).

#### 7.1.2 Timosaponin AIII

Timosaponin AIII (TA-III) interacts with targeted enzymes and cellular pathways to induce ferroptosis in CRC cells. TA-III upregulates genes associated with ferroptosis, such as ACSL4, PTGS2, and FSP1, resulting in increased lipid peroxidation and intracellular lipid ROS levels. These changes lead to oxidative stress and mitochondrial damage, ultimately triggering ferroptotic cell death. Additionally, TA-III induces lipophagy, a form of autophagy that degrades lipid droplets to produce free fatty acids (FFAs). The accumulation of FFAs alters fatty acid oxidation rates and mitochondrial function, further promoting ferroptosis. Moreover, TA-III enhances lysosomal activity through increased Rab7 expression, facilitating lipophagy and elevating unsaturated fatty acid content, exacerbating lipid peroxidation and cell death. In summary, TA-III’s interactions with targeted enzymes and pathways drive ferroptosis, offering potential implications for CRC therapy. The *in vitro* and *in vivo* studies discussed regarding TA-III provide valuable insights into its potential clinical applications for CRC treatment. *In vitro* experiments demonstrate TA-III’s ability to induce ferroptosis in CRC cells by upregulating genes associated with this process, leading to lipid peroxidation, oxidative stress, and mitochondrial damage. Additionally, TA-III promotes lipophagy, further exacerbating cell death. *In vivo* studies using a CRC xenograft model show significant tumor suppression with TA-III treatment, further supporting its efficacy. These findings suggest that TA-III could be a promising therapeutic agent for CRC, potentially offering a novel approach to target cancer cells through the induction of ferroptosis. Further clinical studies are warranted to validate these findings and explore TA-III’s potential as a treatment option for CRC patients. The absence of histological differences in liver and kidney function among the groups suggests that TA-III treatment may not induce significant toxicity in these organs. This finding is crucial as it highlights the potential safety profile of TA-III, which is essential for its translation into clinical applications. It indicates that TA-III treatment may be well-tolerated, minimizing the risk of adverse effects on liver and kidney function. This aspect further enhances the potential of TA-III as a therapeutic agent for colorectal cancer (CRC) treatment, as it suggests a favorable safety profile that could facilitate its clinical development and eventual use in patients (Shen et al., 2024).

## 8 Current limitations

This review, while comprehensive, encounters several limitations that may impact the generalizability and applicability of the findings.

### 8.1 Geographic concentration of studies

A significant portion of the referenced studies were conducted in China. This geographic concentration may limit the applicability of the findings to diverse populations due to potential differences in genetic backgrounds, environmental factors, and dietary habits that influence the efficacy and metabolism of natural products.

### 8.2 Lack of in vivo validation in some studies

Many studies included in this review are preclinical and primarily based on *in vitro* models. While these provide valuable insights into the molecular mechanisms of natural products in inducing ferroptosis in colorectal cancer cells, *in vivo* studies are crucial to evaluate the pharmacokinetics, pharmacodynamics, and therapeutic potential of these products in a biological system. The absence of *in vivo* data may limit the translational value of these findings.

### 8.3 Absence of clinical studies

None of the studies reviewed were clinical trials; thus, the effects observed are limited to preclinical observations. Clinical studies are essential to determine the safety, efficacy, and dosing of natural products in humans, which are critical for advancing any potential therapeutic agent from the bench to the bedside.

### 8.4 Combined effects of natural products

The studies reviewed largely focus on the effects of individual natural products or their components on ferroptosis regulators. However, none investigated the combined or synergistic effects of these natural products when used together. This is particularly relevant in dietary interventions where multiple phytochemicals are consumed simultaneously, potentially interacting in ways that could enhance or inhibit their effectiveness.

## 9 Future perspectives

### 9.1 Exploring combined therapeutic effects

Future research should focus on examining the combined effects of natural products along with standard treatments like chemotherapy and radiotherapy. This integrated approach could reveal synergistic interactions that enhance the efficacy of existing treatments or reduce their side effects. Conducting these studies in clinical settings is particularly critical to transition from theoretical benefits observed in preclinical studies to practical, applicable cancer therapies. Such studies will help determine the optimal dosing, timing, and combination of natural products with conventional treatments, potentially leading to more effective and personalized therapeutic options for colorectal cancer patients.

### 9.2 Nationwide and global studies

To address the geographical bias and enhance the generalizability of findings, future research should include nationwide studies that consider diverse populations. Expanding research beyond specific regions will provide a more comprehensive understanding of how natural products affect different populations globally. This approach will help validate the effectiveness and reliability of natural products as a therapeutic option across different genetic backgrounds and environmental conditions, leading to broader applicability and acceptance of the findings.

### 9.3 Safety profile assessments

Although natural products are generally perceived as safe, rigorous evaluations of their safety profiles are necessary, particularly when used in therapeutic contexts. Future studies should prioritize assessing the toxicity and safety of these compounds *in vivo*, ideally before proceeding to clinical trials. Understanding the potential adverse effects, interactions with other drugs, and long-term safety implications are essential for ensuring the compounds are safe for clinical use. Such safety assessments will also aid in determining acceptable dosage ranges and identifying any contraindications.

### 9.4 Discovery of new ferroptosis regulators

There remains a vast potential to uncover additional natural products that can modulate ferroptosis in ways not yet understood or documented. Future research should aim to expand the scope of the investigation to include lesser-known or newly identified ferroptosis regulators. By exploring untapped natural resources and employing advanced screening techniques, researchers may discover novel compounds with unique mechanisms of action that could lead to groundbreaking treatments for colorectal cancer and possibly other ferroptosis-related diseases. This approach not only broadens the therapeutic arsenal but also deepens our understanding of the complex biology of ferroptosis and its role in cancer pathology.

## 10 Conclusion

This review highlights the current progress in effectively targeting ferroptosis regulators to induce cell death in CRC. Surprisingly, many of these compounds are studied in vivo conditions and xenograft models of mice, demonstrating not only their potential use as anti-cancer products but also their ability to exert less or least toxicity on organs such as the liver, kidney, and heart, making them potential substitutes for conventional chemotherapeutic regimens in colorectal cancers. Amino acid metabolism regulators (GPX4, System xc), iron metabolism regulators (TFRC, ferritin, HO-1, NCOA4), lipid metabolism regulators (ALOXs, ACSL4), and oxidative stress regulators (NRF2, p53, AMPK/mTOR pathway) have all been evaluated and targeted by these small molecules in CRC. These naturally occurring products can also be used in combination with EGFR-TKIs (cetuximab) and chemotherapeutic agents (5-FU, cisplatin), thereby lessening the adverse side effects caused by these chemotherapeutic regimens. Curcumin and Andrographis are both well-studied for their ability to induce ferroptosis by suppressing the mTOR pathway and FSP1 in CRC. Additionally, recent studies have focused on the ferroptosis-inducing ability of butyrates in CRC, introducing them as novel regulators of ferroptosis. Similarly, compounds like Ginsenosides (Rh3, Rh4) are able to induce ferroptosis by modulating oxidative stress regulators (NRF2, ferritinophagy mediators NCOA4) in CRC, providing more targets to effectively induce cell death. To overcome the shortcomings in drug delivery and bioavailability, nanoparticles have surprisingly shown effectiveness in inducing ferroptosis. These nanodrugs, especially those containing Glycyrrhetinic acid and bofutalin, can suppress GPX4 activity in colorectal cancer and cause amino acid disruption, exacerbating ferroptosis. Researchers can also evaluate the synergistic effects of these nanoparticles in the realm of CRC to obtain better tumor inhibitory functions. However, this field is still emerging, and more studies are required to uncover natural products in CRC.
